# Phytotherapy for acute respiratory tract infections in children: a systematically conducted, comprehensive review

**DOI:** 10.3389/fped.2025.1423250

**Published:** 2025-05-01

**Authors:** Wolfgang Kamin, Georg Seifert, Karl Zwiauer, Jan Bonhoeffer, Veerle De Ketelaere, Antonio D'Avino, Jakub Štádler, Juan Carlos Bustamante-Ogando, Ateş Kara

**Affiliations:** ^1^ Department of Pediatrics, Clinic for Pediatrics and Adolescent Medicine, EVK Hamm, Hamm, Germany; ^2^ Department of Pediatrics with a Focus on Oncology and Hematology, Campus Virchow-Klinikum (CVK), Charité—University Medicine Berlin, Berlin, Germany; ^3^ Clinical Department of Pediatrics and Adolescent Medicine, St. Pölten University Hospital, Sankt Pölten, Austria; ^4^ Department of Infectiology and Vaccinology, University Children’s Hospital of Basel, Basel, Switzerland; ^5^ Pediatric Practice, Veldegem, Belgium; ^6^ FIMP (Italian Federation of Primary Care Pediatricians), National Health Care Service, Naples, Italy; ^7^ Children’s Immunology and Allergy Outpatient Centre, MY CLINIC Family Medical Clinic, Prague, Czechia; ^8^ Primary Immunodeficiencies Research Laboratory, National Institute of Pediatrics, Mexico City, Mexico; ^9^ Department of Pediatrics, Pediatric Infectious Disease, Hacettepe University, Ankara, Türkiye

**Keywords:** children, antibiotic resistance, phytomedicine, phytotherapy, complementary therapies, herbal medicine, acute respiratory tract infections

## Abstract

**Background:**

The need to reduce the inappropriate use of antibiotics for the treatment of pediatric acute respiratory tract infections (ARTIs) calls for therapeutic alternatives. The use of herbal medicines (phytopharmaceuticals) to treat ARTIs has increased worldwide. However, the evidence of phytopharmaceutical treatment, especially for children with ARTIs, has not yet been comprehensively presented.

**Objective:**

To identify evidence on the efficacy and tolerability of phytopharmaceuticals in children suffering from ARTIs.

**Methods:**

We searched the literature using two databases (PubMed and Cochrane Library) in accordance with the Preferred Reporting Items for Systematic Reviews and Meta-Analyses (PRISMA) guidelines to identify records of clinical studies investigating the administration of phytopharmaceuticals in children with upper ARTIs (AURTIs) and/or lower ARTIs (ALRTIs).

**Results:**

A total of 45 reports met the eligibility criteria and were included in our review. Of these, only nine reported double-blind, placebo-controlled trials investigating the efficacy and tolerability of phytopharmaceuticals in pediatric ARTIs. Overall, the included reports covered phytopharmaceuticals with the following single active ingredients: various purple coneflower (*Echinacea purpurea L.*) preparations, ivy (*Hedera helix L.*) leaf dry extract, African geranium (*Pelargonium sidoides L*.) root extract (EPs 7630), and pineapple fruit and stem extract preparation. In addition, various reports were included for fixed combination phytopharmaceutical products: eucalyptus oil combinations, five-herb fixed combination extract [BNO 1012 syrup], seven-herb fixed preparation [BNO 1030 solution], thyme herb and ivy leaf extracts, purple coneflower and sage leaf spray, thyme and primrose root extracts, and a preparation containing upper plant parts and roots of purple coneflower, propolis and vitamin C. The most robust efficacy and tolerability evidence available was found for EPs 7630: six double-blind, placebo-controlled trials, eight meta-analyses, four prospective open-label studies, and two observational studies, demonstrating efficacy and good tolerability.

**Conclusion:**

Among the phytopharmaceuticals identified by our systematically conducted, comprehensive literature review, EPs 7630 is supported by sufficient evidence to be regarded as an appropriate treatment to reduce the severity and duration of AURTIs and ALRTIs in children. Regarding other phytopharmaceuticals reported in the literature for the treatment of pediatric ARTIs, further research is needed to close existing evidence gaps.

## 1 Introduction

Pediatric acute respiratory tract infections (ARTIs) are responsible for approximately 70% of antibiotic prescriptions in children (1). Current evidence-based practice guidelines consider almost 50% of these prescriptions unnecessary or problematic (2, 3). Moreover, inappropriate antibiotic treatment of ARTIs in children has contributed to the global problem of antibiotic resistance (4). The European Centre for Disease Prevention and Control (ECDC) endorses antimicrobial stewardship (AMS) as a strategy to help stop the current spread of antimicrobial resistance in Europe (4). The European Academy of Pediatrics (EAP) Curriculum for Common Trunk Training in Pediatrics also recommends that all European physicians practicing in pediatric healthcare should be educated to prescribe antibiotics to children in a rational way (4). Parents also play an important role in antibiotic prescription practices, and clinicians often cite parental misconceptions and expectations for antibiotics as reasons for unwarranted antibiotic prescribing (5–7). To reduce antibiotic expectations, communication within the consultation, prescribing behavior, as well as lay beliefs need to be addressed (8). In this regard, conducting pediatric clinical trials and educating physicians about the rational prescribing of medications to treat RTIs in children are of the utmost importance.

Plants represent potential sources of bioactive compounds such as essential oils, polyphenols, flavonoids, saponins, and alkaloids, which have been reported to relieve symptoms of acute upper respiratory tract infections (AURTIs) such as tonsillitis, tonsillopharyngitis, and rhinitis (9–13) and acute lower respiratory tract infections (ALRTIs) such as acute bronchitis and acute cough (12, 14, 15). Indeed, herbal medicines have traditionally been used to prevent and treat ARTIs and are scientifically valued (16). Phytotherapy is the contemporary definition for using preparations from plants, their parts, or active ingredients prepared from plants, purified and/or standardized using specific pharmaceutical technologies and proper galenic preparations for medicinal use (17). Phytopharmaceuticals are, therefore, final medicinal products with active ingredients derived from plants, parts of plants, or preparations made from plants or parts of plants (18). In accordance with the World Health Organization's (WHO) Traditional Medicine Strategy for 2014–2023, 179 member countries have advocated for the safety, quality, and efficacy of traditional and complementary medicine (19). This includes the correct use of medicinal plants and their derivatives for preventive or curative purposes according to the pharmacological properties of their chemical constituents (20).

Despite the existing scientific recognition of phytopharmaceuticals by regulatory authorities and WHO, many physicians and parents may still consider their medicinal properties to be based on “old wives’ tales” or “folklore” rather than evidence-based medicine (EBM) (21–23). Nevertheless, due to the significant increase in the prevalence of multidrug-resistant bacterial infections in children during the past couple of decades, phytopharmaceuticals have gained a resurgence of interest and popularity among allied health professionals in Western countries and worldwide. Real-world evidence from a retrospective cohort study evaluating 64,836 pediatric outpatients with RTIs showed that phytopharmaceuticals as first-line treatment for acute RTIs significantly reduced antibiotic prescriptions in the further course of the disease (24). Furthermore, a qualitative study from the UK reported that many patients would be willing to try phytopharmaceuticals to treat specific RTI symptoms if advised by their physician, whereas most physicians reported that they would recommend phytopharmaceuticals if evidence-based guidelines were available (25). Without the mention of suitable phytopharmaceuticals in many existing guidelines, a conscious effort is needed to enable physicians to identify, recognize correctly, and position phytopharmaceuticals in the clinical treatment pathway (25).

Education about the indications and uses of phytopharmaceuticals in pediatric RTIs is both reasonable and justified. Continuous critical assessment in accordance with EBM standards is required to demonstrate the efficacy of phytopharmaceuticals in children (26). However, most clinical evidence so far has only been in adults and is thus inadequate to support recommendations of phytopharmaceuticals for preventing or treating ARTIs in children (27, 28). To address this unmet need and identify gaps in existing evidence, we systematically searched the literature for clinical studies evaluating the efficacy and tolerability of phytopharmaceuticals in treating ARTIs in children.

## 2 Methods

### 2.1 Specifications

The Preferred Reporting Items for Systematic Reviews and Meta-Analyses (PRISMA) guideline methodology (29) was used for a systematic search to identify reports on the use of phytopharmaceuticals in the treatment of ARTIs in children. Diseases of interest were the common cold, sore throat, acute bronchitis (AB), acute tonsillopharyngitis (ATP), and acute rhinosinusitis (ARS). Pneumonia, influenza, and obliterative bronchiolitis were not considered under the term ALRTI.

### 2.2 Search strategy

A comprehensive search was conducted using the PubMed and Cochrane Library databases, covering literature from January 1, 2000, to February 21, 2023. Both databases provide comprehensive, high quality reporting according to PRISMA guidelines. To ensure high coverage of the existing literature, a comprehensive search strategy was developed using the following terms in the Title/Abstract: (common cold; upper respiratory tract infection; acute respiratory tract infection; acute bronchitis; acute tonsillopharyngitis; sore throat; acute sinusitis; acute rhinosinusitis); AND (clinical trial; clinical study; randomized; randomised; OR meta-analysis); AND (therapy or treatment); NOT (Covid). No language restrictions were applied. Results were combined and exported to Endnote, where duplicate records and clinical registry records were removed. Manual internet searching was also performed without time period restrictions to identify additional relevant records.

### 2.3 Eligibility criteria and data extraction

Records were single-screened, first by examining the title and second by examining the abstract based on the inclusion/exclusion criteria. The full text report was retrieved and screened if the title or abstract met the inclusion criteria (or if eligibility was unclear). Three independent reviewers applied the eligibility criteria to all the retrieved reports. Full-text reports of systematic reviews with meta-analyses and clinical studies covering randomized trials or non-randomized studies (i.e., prospective cohort and observational studies), with efficacy and tolerability data regarding phytopharmaceuticals in children and/or adolescents with ARTIs, were included. Reports were excluded for the following reasons: abstract only, commentary, narrative review article, no phytopharmaceutical, and adults-only study. Non-English language reports retrieved in full were translated into English for review. Disputes were resolved by discussion. Eligible reports were grouped as relating to AURTIs or ALRTIs and reviewed in full.

### 2.4 Levels of evidence

Individual clinical studies were evaluated in accordance with the Oxford Center for Evidence-Based Medicine 2011 Levels of Evidence (OCEBM) (30). Table 1 shows the adapted version of the OCEBM levels of evidence used.

**Table 1:** Levels of evidence—adapted from Oxford center for evidence-based medicine (OCEBM) 2011 (30).

Level	Evidence (Treatment benefits)
I	Systematic review or meta-analysis of RCTs, high-quality individual RCTs
II	Systematic review or meta-analysis of cohort studies, low-quality individual RCTs, prospective studies
III	Systematic review of case-control studies, retrospective cohort studies, observational study
IV	Case series
V	Expert opinion

RCTs, randomized controlled trials.

### 2.5 Grade of recommendation

Grades of recommendation for included phytopharmaceuticals were assigned according to the strength of evidence defined in Table 2. For example, to obtain the highest grade of recommendation (Grade A), consistent Level 1 studies had to be available.

**Table 2:** Grades of recommendation—adapted from Oxford center for evidence-based medicine (OCEBM) 2011 (30, 85).

Level	Evidence (Treatment benefits)
A	Consistent Level 1 studies
B	Consistent Level 2 or 3 studies or extrapolations from Level 1 studies
C	Level 4 studies or extrapolations from Level 2 or 3 studies
D	Level 5 evidence or troublingly inconsistent or inconclusive studies of any level

## 3 Results and discussion

### 3.1 Included reports

**Figure 1:**
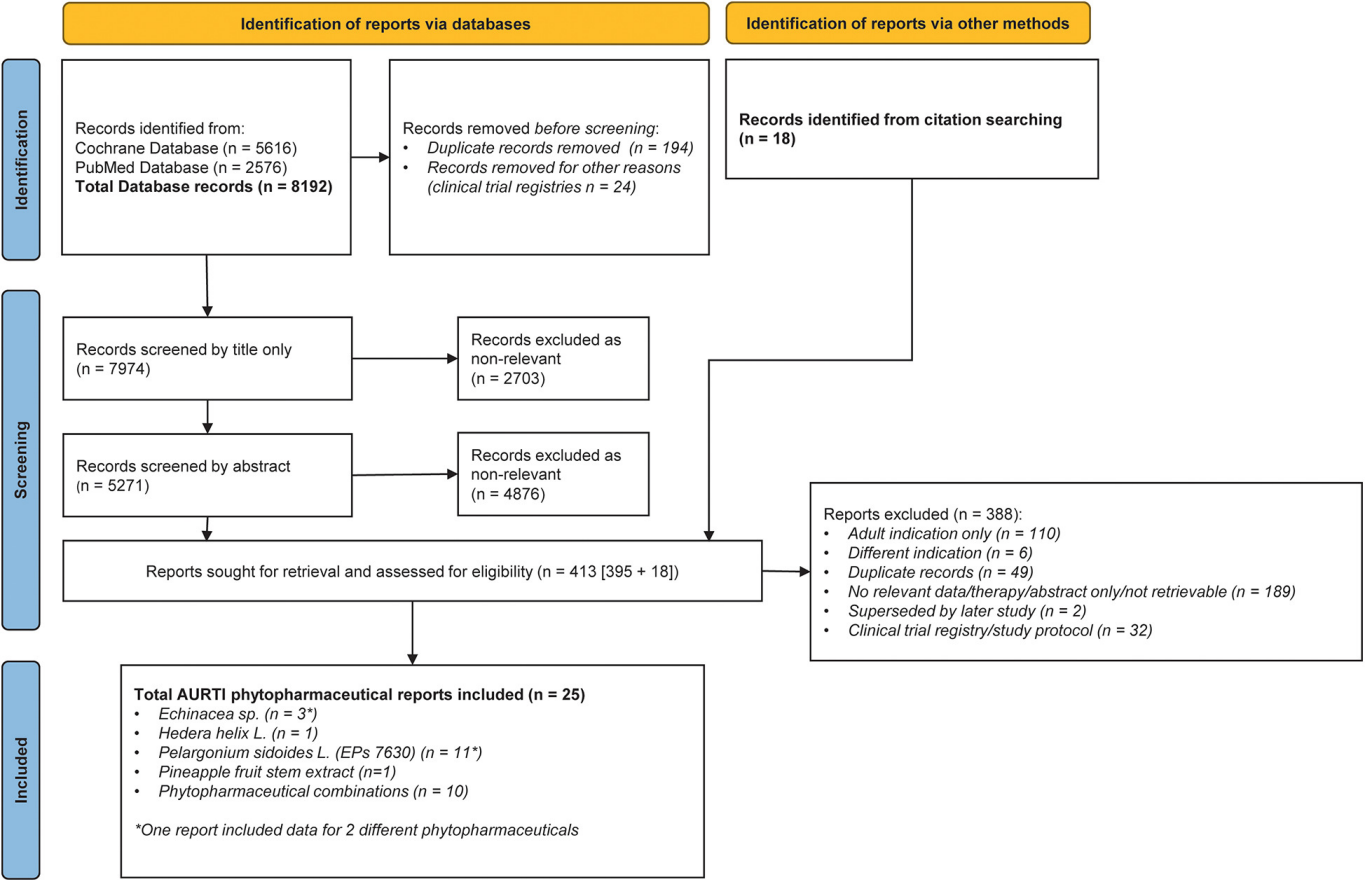
PRISMA diagram of reports relating to pediatric acute upper respiratory tract infections (AURTIs).

**Figure 2:**
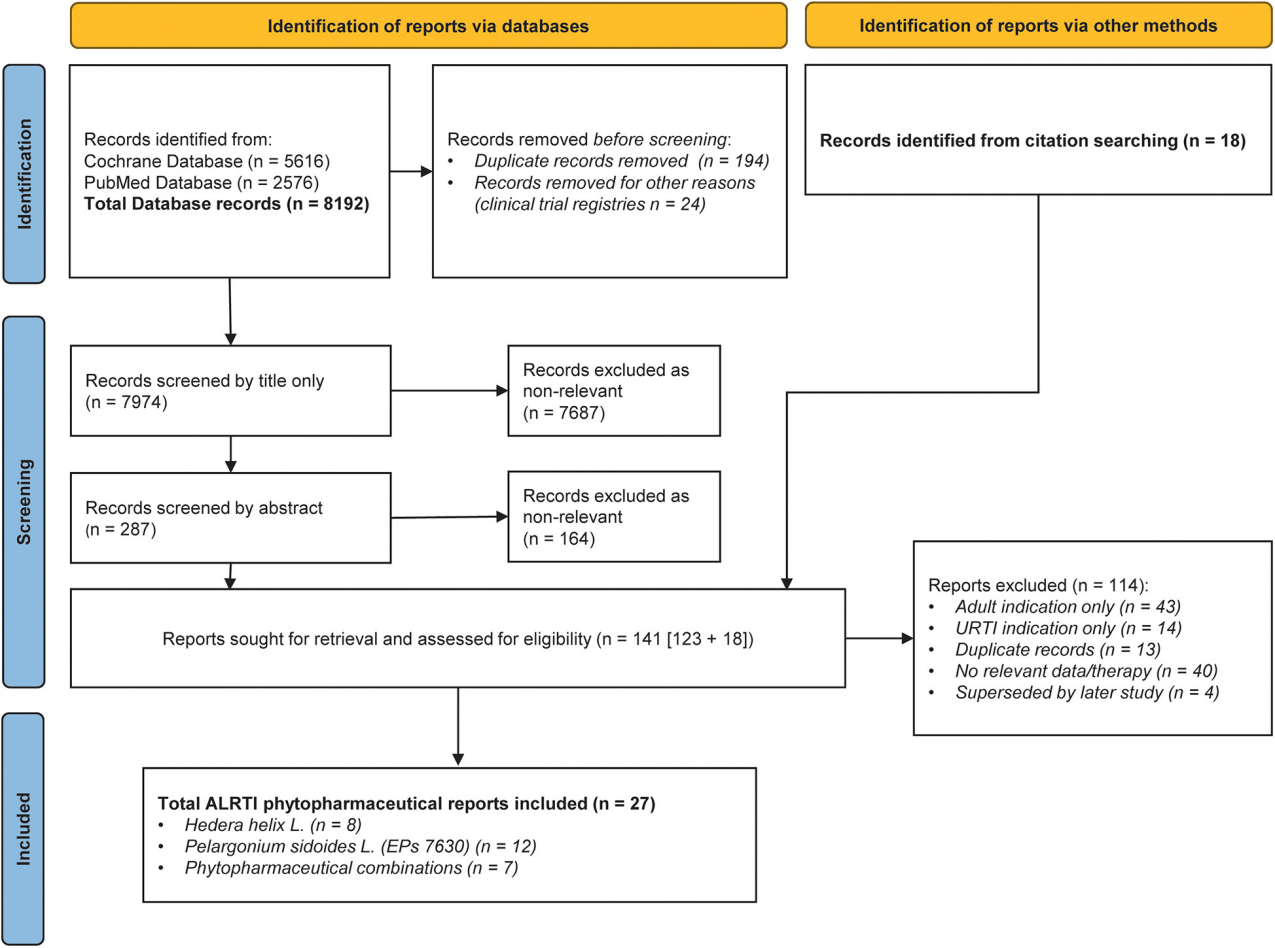
PRISMA diagram of reports relating to pediatric acute lower respiratory tract infections (ALRTIs).

The database searches identified 8,192 database records. Of these, 218 were duplicates or removed for other reasons, resulting in 7,974 records to be screened by title and abstract. An additional 36 records (18 for AURTIs and 18 for ALRTIs) were identified from manual citation searching in parallel. Overall, 554 (413 for AURTIs and 141 for ALRTIs) reports were reviewed in full for eligibility and grouped according to phytopharmaceutical. Flowcharts of the literature search are depicted in Figures 1, 2.

For AURTIs, a total of 25 reports investigating the efficacy and tolerability of phytopharmaceuticals with single active ingredients or combinations in children met the eligibility criteria and could therefore be included for evidence evaluation (Table 3): one systematic review and meta-analysis of five relevant placebo-controlled, randomized controlled trials (RCTs) of which two were relevant in AURTIs (31)—this report was included twice for two different phytopharmaceutical groups; one systematic review with two relevant RCTs included in a meta-analysis (32); four meta-analyses of double-blind, placebo-controlled trials (12, 13, 33, 34); seven double-blind placebo-controlled trials (35–41); one triple-parallel group randomized trial (42); one single-blind, placebo-controlled randomized trial (43); five open-label randomized controlled trials (44–48); two prospective open-label studies (49, 50); two observational studies (51, 52); and one case-controlled study (53).

**Table 3:** Summary of phytopharmaceutical reports and level of evidence for AURTIs in children.

Author, year published	Type of report	Pediatric patients, *N* (age range)	Indication	Treatments (dose)	Primary endpoint key outcome measure	Key efficacy/safety results	Level of evidence
Preparations from *Echinacea purpurea L. (purple coneflower) aerial parts, seeds, and/or root extract*							
Anheyer et al. 2018 (31)	Systematic review and meta-analysis of 11 RCTs in pediatrics (4 studies investigated the effectiveness of *Echinacea purpurea L.*)	958 (for the 4 RCTs of *Echinacea purpurea L.*) (1-11 years)	Common cold, acute otitis media, unspecified acute RTI	*Echinacea purpurea L.* various forms (including dried herb extract; liquid extract of the fresh roots and dried mature seeds or 80 ml juice from fresh flowers) or a combination of *Echinacea purpurea L.* (above ground plant parts) and *Echinacea augustifolia L.* (roots) within a compound herbal extract in liquid form	Various outcome measures for individual RCTs	Due to the heterogeneity of outcome measures and control conditions, no meta-analysis could be performed for the studies presented.	I
Spasov et al. 2004 (42)	Triple parallel group RCT	133 (4–11 years)	URTI: common cold	Group 1: usual treatment plus a fixed herbal combination containing standardized *Andrographis paniculata* N. extract (2 × 3 capsules/day) (*n* = 53); Group 2: usual treatment plus *Echinacea purpurea L.* extract 80 ml (3 × 10 drops/day) (*n* = 41); Group 3: usual treatment (*n* = 39)	Efficacy parameters measured by clinical records: quantitative parameters (amount of nasal secretions over 24 h, increase in weight of soft tissues used (g), mucociliary clearance (g), rhinoflowmetry (ml/second); immunological parameters	Positive change in the URTI in all three groups; group 1 recovered quicker than the other groups; by day 4, results showed that group 1 had a pronounced effect on nasal secretion; group 2 had a significant effect only on day 8 compared to group 3, the difference between groups 1 and both 2 and 3 was significant from day 5 and day 4; similarly, the difference between group 1 vs. 2 and 3, with respect to mucociliary clearance was significant at the third visit; erythrocyte sedimentation rate and leukocyte count measured at day 8 showed a significant difference in leukocyte count between group 1 and group 3, there was no difference between group 1 and 2.	II
Taylor et al. 2003 (36)	Double-blind, placebo-controlled RCT	407 (2‒11 years)	URTIs	*Echinacea purpurea L. above-ground plant parts*: children 2–5 years received 7.5 ml/day (3.75 ml twice a day) during a URTI, and 6–11 years 10 ml/day (5 ml twice a day). vs. placebo.	Duration and severity of symptoms and adverse events recorded by parents	Median duration of URTIs was 9 days [95% CI: 8, 10 days]. There was no difference in duration between URTIs treated with echinacea or placebo (*p* = 0.89). There was also no difference in the overall estimate of severity of URTI symptoms between the two treatment groups (median, 33 in both groups; *p* = 0.69). *Echinacea purpurea L.*, as dosed in this study, was not effective in treating URTI symptoms in children aged 2–11 years.	I
Hedera helix L. leaf extracts							
Strauss-Grabo et al. 2011 (51)	Observational study	310 (11–85 years)	Common cold and cough	Ivy leaf dry extract; 2 × 25 mg tablets twice daily for 7 days	Tolerability and safety rated by means of questionnaires	Ivy leaf dry extract tablets were reported to have good to very good tolerability by both the practitioners (98.5%) and patients (96.4%), with high acceptance and compliance (rated as “good” in 98.8% of all cases).	III
Pelargonium sidoides L. root extract (EPs 7630)							
Kamin et al. 2022 (33)	Meta-analysis of 2 double-blind, placebo-controlled RCTs in pediatric ATP^a^	267 (6‒10 years)	ATP, sore throat	EPs 7630 (*n* = 133) 20 drops 3× daily for 6 days vs. placebo (*n* = 134)	Change in symptom severity of sore throat according to the TSS from baseline to day 4; Complete remission of sore throat until day 4	See outcomes for ATP in Bereznoy et al. and Berezhnoy et al. (35, 40). Children treated with EPs 7630 showed a significantly pronounced improvement of the symptom “sore throat” after 4 days compared to placebo treatment [mean difference—0.93; 95% CI: −1.14, −0.72]. A significant advantage of EPs 7630 was shown for the number of patients with complete remission of the symptom “sore throat” by Day 4 [relative risk 2.00; 95% CI: 1.35, 2.97].	I
Yao et al. 2022 (34)	Meta-analysis of 30 RCTs (3 in pediatric acute non-streptococcal tonsillopharyngitis; 2 in pediatric URTIs)^b^	434 (for the 5 RCTs in ATP *n* = 345 [mean age 7.4 ± 1.2–8.0 ± 1.0] and URTIs *n* = 89 [1–14 years]	ATP, common cold, URTIs	EPs 7630: for the ATP trials (*n* = 173) 3 × 20 drops/day (or day 1–2: 20 drops hourly while awake; day 3–6: 3 × 20 drops) for 6 days vs. placebo (*n* = 172); EPs 7630 for the URTI trials (*n* = 44) 3 × 10 drops daily for 7 days vs. placebo and 3 × 10 drops (1–5 years), 3 × 20 drops (6–12 years) or 3 × 30 drops (>12 years daily) for 5 days) or paracetamol tablets as best supportive care (*n* = 45)	Change of TSS on day 7 for 3 acute non-streptococcal studies; Change of total CIS on day 7 and the improvement rate of nasal symptoms for 2 URTI studies	See outcomes for ATP in Bereznoy et al., Berezhnoy et al. and Timen et al. (35, 39, 40). Pooled results for the two URTI pediatric studies showed that the flavonoids group significantly increased the improvement rate of nasal symptoms compared to the control group [RR 2.51; 95% CI: 1.42, 4.42; *I*^2^ = 0; *p* = 0.513].	I
Seifert et al. 2021 (13)	Meta-analysis of 3 double-blind, placebo-controlled RCTs in children with ATP^b^	345 (6–10 years)	ATP	EPs 7630 (*n* = 173): 3 × 20 drops/day (or day 1–2: 20 drops hourly while awake; day 3–6: 3 × 20 drops) for 6 days vs. placebo (*n* = 172)	Cumulative paracetamol administration; inability to attend school at treatment end	See outcomes for ATP in Bereznoy et al., Berezhnoy et al. and Timen et al. (35, 39, 40). Compared with placebo, EPs 7630 reduced the cumulative paracetamol dose by an average of 449 mg [95% CI: 252, 646 mg; *p* < 0.001]. A total of 19.1% (EPs 7630) and 71.5% (placebo) of children were still unable to attend school at the end of the treatment [risk ratio = 0.28; 95% CI: 0.16, 0.48; *p* < 0.001].	I
Seifert et al., 2019 (12)	Meta-analysis of 6 double-blind, placebo-controlled RCTs (3 trials in ATP and 3 in AB)^b^	345 (6–10 years)	ATP (*n* = 345) [or AB for acute LRTIs (*n* = 178)]	EPs 7630 (*n* = 173): 3 × 20 drops/day (or day 1–2: 20 drops hourly while awake; day 3–6: 3 × 20 drops) for 6 days vs. placebo (*n* = 172)	Cumulative paracetamol administration; inability to attend school at day 6/7	See outcomes for ATP in Bereznoy et al., Berezhnoy et al. and Timen et al. (35, 39, 40). EPs 7630 reduces the severity and duration of disease-associated symptoms in children with non-streptococcal ATP and AB. Children treated with EPs 7630 required less paracetamol co-medication and were able to return to school earlier. EPs 7630 ameliorated the unpleasant effects of the febrile response, achieved symptomatic improvement and accelerated the restoration of normal functioning without potentially counterproductive antipyresis, also reducing patient risk by avoiding the potentially harmful side effects of paracetamol.	I
Anheyer et al. 2018 (31)	Systematic review and meta-analysis of 5 RCTs in pediatrics (5 studies in the meta-analysis investigated the effectiveness of *Pelargonium sidoides L.; 1 trial in tonsillitis, 1 trial in ARTI plus 3 AB trials included in the meta-analysis*)	171 (tonsillitis trial *n* = 173 [mean age 7.6 ± 1.3 years] and ARTI trial *n* = 28 [mean age 30.1 months, range 12–60 months]	Tonsillitis (1 trial; *n* = 173); ARTI with transient hypogammaglobulinemia (1 trial; *n* = 28)	Tonsillitis: EPs 7630 20 drops (80 mg) 3× day vs. placebo for 6 days; ARTI: EPs7630 (ratio 1:8–10; extraction solvent: ethanol 11%) 3 × 10 drops/day for 7 days	Tonsillitis: Change in TSS from baseline to day 4; Evaluation of symptoms using a questionnaire	The meta-analysis of 5 studies (including 1 one tonsillitis trial, one ARTI trial and 2 AB trials) showed evidence for the efficacy of EPs 7630 in treating URTI and LRTIs symptoms [RR = 2.56; 95% CI: 1.54, 4.26; *p* < 0.01; heterogeneity: *I*^2^ = 38%; *χ*^2^ = 9.63; *p* = 0.14; NNT = 8; 95% CI: 5, 20] compared to placebo. Meta-analysis of 4/5 studies investigated the safety/tolerability of EPS 7630. Compared to placebo, no differences between the groups with regard to reported adverse events were observed [RR = 1.06; 95% CI: 0.42, 2.66; *p* = 0.9; heterogeneity: *I*^2^ = 72%; *χ*^2^ = 10.64; *p* = 0.01]. Moderate evidence for the efficacy and safety in the treatment of RTIs in children was reported. No separate analysis was performed for URTIs.	I
Timen et al. 2015 (39)	Double-blind, placebo-controlled, multicentre RCT	78 (6‒10 years)	ATP	EPs 7630 *n* = 40 (Day 1: 20 drops/hour while awake, Day 3‒6: 30 × 20 drops) vs. placebo *n* = 38 for 6 days	TSS (7 symptoms) ≤4 points at day 4	Success rates of 90.0% in the EPs 7630 and 44.7% in the placebo group (*p* < 0.001). The analysis indicated highly significant advantages of EPs 7630 (*n* = 40) over placebo (*n* = 38) regarding all signs and symptoms typical of ATP, e.g., for the total score of objective and subjective symptoms on days 2, 4 and 6 (*p* < 0.001). EPs 7630 is effective in treating children with ATP and very well tolerated.	I
Bereznoy et al. 2016 (40)	Double-blind, placebo-controlled RCT	124 (6‒10 years)	ATP	EPs 7630 (20 drops 3× day) vs. placebo for 6 days	Change in TSS on day 4 compared to baseline	After 4 days of treatment, the TSS was decreased from 9.6 ± 1.2–2.8 ± 2.6 points in the active medication group and from 9.5 ± 1.3–6.1 ± 4.1 points in the placebo group (*p* < 0.001), indicating superiority of EPs 7630 over placebo.	I
Schapowal and Heger 2007 (49)	Prospective, open-label, non-controlled multicenter study	361 (1–94 years)	Acute sinusitis	The regular study period for patients with acute sinusitis was 28 days; patients with chronic recurring sinusitis received eight additional weeks of recurrence prophylaxis following 28 days of treatment with EPs 7630. EPs 7630 solution was prescribed as follows: for acute sinusitis: 30 drops once per hour (max. 12× per day) in the first 1–2 days, thereafter 30 drops 3× per day; children under 12 years of age: 20 drops once per hour (max. 12× per day) in the first 1–2 days, thereafter 20 drops 3× per day; for chronic recurring sinusitis within the scope of recurrence prophylaxis: 2 × 30 drops per day for adults and 2 × 20 drops per day for children under 12 years	Change in objective and subjective symptoms	80.9% of the participants reported freedom from symptoms or a significant improvement of symptoms at day 28. Therapy successes (remissions or improvements) were recorded in more than 90% of the participants within the first four weeks for all individual symptoms according to the symptom score. 17 patients reported adverse events for which a connection with the study medication could not be ruled out, but was considered “unlikely”; in most cases, these were gastrointestinal symptoms. A separate effectiveness analysis in children was not conducted; however, the health-compliant questionnaire (HCQ-5) score decreased from an initial 14.2 points to 6.8 points after 28 days in a subgroup of children (*n* = 30)	III
Bereznoy et al. 2009 (50)	Prospective, multicenter, open-label study	1,000 (2–35 years)	ATP or chronic/recurrent tonsillitis	EPs 7630 (2–6 years: 3 × 10 drops/day; >6–12 years: 3 × 20 drops/day; >12 years-adult: 3 × 30 drops/day for 7 days	Change in mean total ATS score (7 symptoms) on day 7 or day 35 in patients with chronic/recurrent tonsillitis	Mean total ATS score declined by 10.5 ± 4.7 points; the responder rate by Day 7 was 88.2%. A total of 94.7 to 98.2% of patients showed a remission and/or improvements in the following individual symptoms: difficulty swallowing, sore throat, salivation, redness, coating/detritus left and right, and fever. The outcome of therapy by Day 7 was described as “free of complaints” or “noticeably improved” by 80.9% of the investigators/patients. The study medication was very well tolerated.	III
Bereznoy et al. 2003 (35)	Double-blind, placebo-controlled RCT	143 (6‒10 years)	ATP	EPs 7630 (20 drops 3× day) vs. placebo for 6 days	Decrease in TSS from baseline (day 0) to day 4	Decrease in TSS from baseline (day 0) to day 4 was 7.1 ± 2.1 points under EPs 7630 (*n* = 73), and 2.5 ± 3.6 points under placebo (*n* = 70)	I
Blochin and Heger (48)	Prospective, open, single-center, randomized pilot study	60 (6–10 years)	Acute tonsillitis	EPs 7630 (20 drops 3× day) vs. symptomatic treatment (gargling with fruit vinegar [1 dessertspoonful in 1 L of warm water] and cold wet compresses: every 2 h until improvement and then 3× day) for 10 days	Change in TSS, evaluated on a 4-point scale; reduction in total TSS to ≤4 points (prospectively defined); change in the individual TSS and other symptoms evaluated on a 4-point scale; tolerance of the treatment was evaluated by the investigator and the patient on a 4-point rating scale	By day 2 of treatment, tonsillitis symptoms were improving more rapidly in the EPs 7630 group than the symptomatic group. On day 4 of treatment, the response, defined as total score for typical tonsillitis symptoms ≤4 points, was greater in the EPs 7630 group than in the group on symptomatic treatment (*p* = 0.001, Fisher's two-tailed exact test, exploratory). No adverse events occurred during the study. All patients finally evaluated the treatment with EPs 7630 as very well or well tolerated.	III
Pineapple fruit and stem extract							
Guo et al. 2006 (32)	SR of 10 RCTs and meta-analysis (≥4 RCTs included children, 2 of which were included in the meta-analysis)	99 in 2 double-blind RCTs (9 years–74 years)	Acute rhinosinusitis	Various pineapple stem extract (meta-analysis of 2 RCTs in children)	Assessment of the efficacy of herbal preparations for treating rhinosinusitis vs. placebo	Meta-analysis of the 2 double-blind RCTs in acute sinusitis suggested that adjunctive use of pineapple extract significantly improves some symptoms of ARS; results could not be separated for children, adolescents and adults.	I
Phytomedicine combinations							
Eucalyptus oil combinations:							
Karpova et al. 2016 (52)	Observational study	60 (6‒10 years)	Acute rhinosinusitis	Eucalyptus oil combined with lemon/orange/myrtle oil [monoterpenes (orange oil)/d-limonene (lemon oil)/1,8-cineole (eucalyptus oil)/alpha-pinene (myrtle oil)] (120 mg 3× day for 7 days) (*n* = 30) in addition to conventional therapy vs. conventional therapy (symptomatic, irrigation) or systemic antimicrobials (*n* = 30)	Treatment effectiveness based on clinical examination, including rhinoendoscopic examination on days 3, 7 and 14 after start of treatment and 4-point VAS completed by parents of children	VAS scores reflecting the severity of rhinorrhoea, nasal congestion and cough were significantly different (*p* < 0.05) in the observation groups in severity of symptoms on days 7 and 14 after the start of treatment, and in the dynamics of nasal congestion reliable difference between patients of the observation, groups were noted on day 3 of treatment. The duration of nasal vasoconstrictors as a symptomatic therapy was reduced in the eucalyptus oil combination group (2.2 ± 0.4 days) vs. the control group (3.6 ± 0.5 days). No undesirable side effects were associated with the use of the eucalyptus/lemon/orange/myrtle oil combination. The use of eucalyptus/orange/lemon/myrtle oil for uncomplicated ARS in children is clinically highly effective, convenient and safe.	III
Paul et al. 2010 (38)	Partially double-blind, placebo-controlled RCT	138 (2‒11 years)	URTI and nocturnal cough	Ointment with a combination of camphor, menthol, and eucalyptus oil (5 ml 2–5 years and 10 ml 6–11 years) vs. petrolatum ointment and no treatment for one single night; total study duration was two consecutive nights	Parent-reported outcome surveys of symptom relief	The ointment with camphor, menthol and eucalyptus oil was superior over petrolatum rub ointment for cough severity, child and parent sleep difficulty, and combined symptom score but not for rhinorrhea. The ointment with camphor, menthol and eucalyptus oil was not better than “no Treatment” for any outcome. In the with camphor, menthol and eucalyptus oil ointment group, 46% reported at least one adverse event vs. 0% in the petrolatum ointment and “no Treatment” groups; major events in the with camphor, menthol and eucalyptus oil ointment group were burning sensation of the skin and eyes.	II
Echinacea extract combination [from an extract of purple coneflower aerial parts and root extract, propolis and vitamin C]:							
Cohen et al. 2004 (41)	Double-blind, placebo-controlled, multicentre RCT	328 (1–5 years)	Acute RTIs	A preparation containing an extract of 50 mg/ml of echinacea (upper plant parts of *E. purpurea* and roots of *E. angustifolia*), 50 mg/ml of propolis, and 10 mg/ml of vitamin C for 12 weeks vs. placebo. Dosage was 2× daily: 5.0 ml for 1–3 years and 7.5 ml for 4–5 years. If an episode of acute illness occurred during the study, the dosage was increased to 5.0 ml and 7.5 ml, respectively, 4× daily during the episode only.	Efficacy evaluation: total no. episodes, total no. of illness days, % children with ≥1 episodes, no. episodes per child, days of illness per child and duration of individual episodes.	The number of children who experienced 1 or more respiratory tract illness episodes during the 12 weeks of the study, the total number of episodes, and the mean number of episodes per child were significantly lower in the *E.purpurea* group than the placebo group [by 43% [*p* < 0.01], 55%, and 50% [*p* < 0.001], respectively]. The total number of illness days and duration of individual episodes were also significantly lower in the echinacea extract group compared to placebo. Similar findings were noted when the children 3 years and younger and those older than 3 years were analyzed separately (data not shown).	II
2 herb preparation [from a mixture of purple coneflower extract (aerial parts and root) and sage leaves]:							
Schapowal et al. 2009 (37)	Multicentre, double-blind, double-dummy RCT	154 (adolescents ≥12–18 years and adults)	Sore throat	2-herb preparation *with aqueous alcoholic (57.3% m/m ethanol) fresh-plant extract from purple coneflower herb 95% aerial parts and 5% root)*/sage leaves spray 50 ml (*n* = 80) vs. 1% chlorhexidine gluconate 2% lidocaine hydrochloride spray (=74), 2 puffs every 2 h up to a maximum of 10× daily	Comparison of the response rates of the two treatment groups after the first, second, and third day	The 2 herb preparation spray exhibited similar efficacy to the chlorhexidine/lidocaine spray treatment in reducing sore throat symptoms during the first 3 days (*p* = 0.5083) in adolescents and adults (no separate analysis available). Response rates after 3 days were 63.8% in the 2 herb preparation group and 57.8% in the chlorhexidine/lidocaine group (adolescents and adults combined data only, no separate analysis).	II
3 herb preparation [from a mixture of ivy leaf dry extract, marshmallow root and hedge mustard seeds]:							
Khan et al. 2019 (53)	Case-control phase 2 study	60 (10–60)	Acute cough, common cold	3-herb preparation (from a mixture of Ivy leaf dry extract, marshmallow root and hedge mustard seeds) chewable tablets for 15 days (*n* = 30 test group) vs. control group (*n* = 30)	The safety and effectiveness using the Leicester cough questionnaire (LCQ) assess cough-related QOL questions (quality of life).	At 95% CI, *p*-value was *p* ≤ 0.005 for cough variables, including cough bouts, sputum viscosity, chest congestion, sore throat and shortness of breath. The LCQ cough scale score was higher in the test group as compared to control group. The test group also showed good tolerability in terms of palatability.	III
Khan et al. 2018 (43)	Single-blind, placebo-controlled RCT	150 (3–>15 years)	Acute and chronic cough, common cold, flu symptoms	3-herb preparation (from a mixture of ivy leaf dry extract 70 mg, marshmallow root 1,000 mg and hedge mustard seeds 100 mg) per 10 ml, 3× daily vs. placebo	The combined effect of the granules on symptoms of cough (acute LRTIs), common cold and flu (acute URTIs); safety and efficacy	Cough granules of a three herb preparation showed improvement in sneezing, stuffy nose, sore throat/chest discomfort, fatigue/weakness, post nasal drip aches/pain, each *p* < 0.0001 (treatment vs. placebo).	II
5 herb extract [prepared from extracts of primrose flowers/gentian root verbena leaves elder flowers common sorrel herb]:							
Sen’kevich et al. 2021 (44)	Open-label, prospective comparative RCT	107 (4‒5 years)	Acute RTIs, acute rhinosinusitis	Three groups: Group 1: 5-herb extract combination (primrose flowers/gentian root verbena leaves elder flowers common sorrel herb) oral drops age-appropriate doses 3× day for 14 days; Group 2: elimination irrigation and topical decongestants plus fluimucil antibiotic age-appropriate doses for 14 days; Group 3: elimination and irrigation therapy and topical decongestants for 14 days	Parent and physician assessment of progression of clinical symptoms (reduction in rhinitis, recovery of nasal breathing, sleep and appetite, disappearance of earaches and cough, normalization of body temperature) and related treatment in 3, 7, 10 or 14 days compared to baseline.	5 herb extract combination treatment of children with acute viral rhinosinusitis contributes to a more pronounced positive symptoms’ dynamics than children who did not receive the fixed herbal combination. Treatment with 14 days of age-appropriate doses of the herbal medicine combination resulted in a notable decrease in nasal congestion, a reduction in nasal discharge, and a decrease in the quantity of blocked nasal passages.	II
Popovych et al. 2020 (45)	Open-label, multicentre, comparative RCT	292 (6‒11 years)	Acute rhinosinusitis	5-herb preparation (extract BNO 1012 syrup), [3.5 ml 3× day plus nasal irrigation 4×day plus standard symptomatic therapy] or standard therapy for 10 days	Reduction in the sinusitis severity according to a 4-point medical assessment scale (MSS) (nasal congestion, rhinorrhea, post-nasal drip) at each visit, dynamics of self-scoring of rhinorrhea and headache (according to a 10-point VAS), “therapeutic benefit” in days, frequency of antibiotic prescriptions	BNO 1012 syrup, in addition to the standard symptomatic treatment of AR, provides a clinically significant, adequate reduction in the severity of rhinorrhea, nasal congestion and post-nasal drip, assessed by a physician at Visit 2 (*p* < 0.005). Significant differences are noted in the patient's self-scoring of rhinorrhea on the second or third day in viral rhinosinusitis and from the fourth to the eighth day in post-viral rhinosinusitis.	II
Popovych et al. 2018 (47)	Prospective, open-label, multicentre RCT	184 (6‒11 years)	Acute rhinosinusitis	5-herb extract preparation (primrose flowers/gentian root/verbena leaves/elder flowers/common sorrel herb) syrup (3.5 ml 3× day in combination with therapeutic irrigation 4× day to standard therapeutic irrigation) for 10 days plus age-specific doses of symptomatic medications such as paracetamol, decongestants	Improvements in symptoms	The 5 herbal preparation syrup effectively alleviates the symptoms of acute viral rhinosinusitis in children. Furthermore, the prescription of antibiotics was also reduced.	II
7 herb extract [prepared from a mixture of horsetail herb yarrow herb marshmallow root, walnut leaves, dandelion plant greens chamomile herb/oak bark]:							
Popovych et al. 2019 (46)	Open-label, multicentre, comparative RCT	238 (6‒18 years)	Acute non-bacterial tonsillitis	7-herb extract (prepared from a mixture of horsetail herb yarrow herb marshmallow root, walnut leaves, dandelion plant greens chamomile herb/oak bark) as a supplement to standard symptomatic therapy [benzydamine hydrochloride] vs. standard therapy; (7 herb extract oral drops in the following doses: in acute disease manifestations, first 5 days: 6–11 years received 15 drops 6× day; ≥12 years 25 drops) 6× day. After alleviation of acute disease manifestations (days 5–10): 6–11 years received 15 drops 3× day; ≥12 years 25 drops 6× day	Sore throat dynamics at rest and swallowing, throat irritation associated with cough, general condition, day of withdrawal of antipyretics, the share of treatment responders, as well as assessment of “therapeutic benefit” from the use of the 7 herb extract	With the 7 herb extract, there was a decreased intensity of acute tonsillitis symptoms to 1 point and lower, assessed using a 4-point scale starting from day 5 of treatment (*p* < 0.005), alleviation of local symptoms and general condition starting from day 2 of the disease (*р* < 0.001), withdrawal of antipyretics starting from day 4 of treatment (*p* < 0.005), an increase of the number of treatment responders to 81.6% (*p* < 0.005) vs. the control were reported. “Therapeutic benefit” was 4.2 days. All patients tolerated phytotherapy well, and no adverse reactions were seen.	II

AB, acute bronchitis; AE, adverse event; AOM, acute otitis media; ARS, acute rhinosinusitis; ATP, acute tonsillopharyngitis; CI, confidence interval; Cineole, eucalyptol; CIS, cold intensity score; LRTI, lower respiratory tract infection; 7 herbal preparation, *Equisetum arvense L.* (horsetail) herb *Achillea millefolium L.* (yarrow) herb *Althaea officinalis L.* (marshmallow) root *Juglans regia L.* (walnut) leaves *Taraxacum officinale FH Wigg*. (dandelion) plant greens *Matricaria recutita L*. (chamomile) *Quercus robur L.* and *Quercus pubescens Willd (oak bark)*; NNT, numbers needed to treat; OMT, osteopathic manipulative treatment; OR, odds ratio; RCT, randomized controlled trial; RTI, respiratory tract infection; Five-herb combination and BNO 1012 syrup extract, *Primula veris L*. (primrose) flowers *Gentiana lutea L.* (gentian) root Verbena officinalis folium (verbena) leaves *Sambucus nigra L*. (elder) flowers *Rumex acetosa L.* (common sorrel) herb; TSS, Tonsillopharyngitis Severity Scale; URTI, upper respiratory tract infection; VAS, visual analog scale; WMD, weighted mean difference; y, years.

^a^ Includes the same 2 RCTs (Bereznoy et al. and Bereznoy et al.) (35, 40).

^b^ Include the same 3 RCTs (Timen et al., Bereznoy et al. and Bereznoy et al.) (35, 39, 40).

For ALRTIs, a total of 27 reports on studies investigating the efficacy and tolerability of phytopharmaceuticals with single active ingredients or combinations in children met the eligibility criteria and could therefore be included for evaluating evidence (Table 4): one systematic review and meta-analysis of 11 RCTs of which three double-blind placebo-controlled trials were relevant (54); one systematic review and meta-analysis of eight double-blind, placebo-controlled studies of which three were relevant (55); one systematic review and meta-analysis of five double-blind, placebo-controlled RCTs of which three were relevant (31); one meta-analysis of six RCTs that included three relevant RCTs (12); one meta-analysis of 7 RCTs of which three RCTs included children (34); one double-blind active comparator RCT (56); one Cochrane review and meta-analysis of three double-blind, placebo-controlled trials (57); five double-blind, placebo-controlled randomized trials (38, 41, 58–60); one open-label active-controlled RCT (61); one single-blind, placebo-controlled RCTs (62); four prospective studies (63–66); eight observational studies (51, 67–73); and one case-controlled study (53).

**Table 4:** Summary of phytopharmaceutical reports and level of evidence for ALRTIs in children.

Author, year published	Type of report	Pediatric patients, *N* (age range, years)	Indication	Treatments (dose)	Primary endpoint key outcome measure	Key efficacy/safety results	Level of evidence
Hedera helix L. leaf extracts							
Kruttschnitt et al. 2020 (63)	Prospective, non-interventional cohort study	139; 30 children [≤13 years (*n* = 20 children); 14–17 years (*n* = 10 children)]	Acute bronchitis	Ivy leaf extract cough syrup *n* = 28 children or acetylcysteine (ACC) monotherapy; granules, syrup, or effervescent tablet) *n* = 2 children	Change from baseline BSS to score at Day 7	Comparable improvement in both groups for all assessments except dyspnoea and number of cough attacks, which showed a higher improvement in the ivy leaf extract group compared with ACC. Both physicians and patients described the efficacy of ivy leaf extract as comparable with ACC. Observations of tolerability were comparable for both products.	III
Olszanecka-Glinianowicz et al. 2020 (68)	Observational study	5,162 (2–5 years)	Productive cough	Ivy leaf dry extract (*Hedera helix L*., folium) (4–8:1)—8.25 mg/1 ml, (extraction solvent: ethanol 30% m/m) syrup administered twice a day	Severity of cough and satisfaction of the child's guardian with the treatment	During observation, the percentage of children with intensive, very common night and affected daily activities due to cough decreased significantly; 68.2% of children's guardians were very satisfied with the effect.	III
Schönknecht et al. 2017 (72)	Open-label, non-interventional, non-randomized study	464 (2–12 years)	Productive cough	Ivy leaf extract 33 mg/4 ml for 7 days	Effectiveness and safety (assessed by BSS, temperature, and prescription of antibiotic therapy)	Improvement in cough 93.3%; improvement in chest pain on coughing 84.7%, in wheezing 90.0%, in dyspnoea 88.7%, in auscultation changes in 94.8%, decline or normalization of body temperature in 96.0% of subjects. The non-antibiotic treated group showed comparable proportions.	III
Lang et al. 2015 (67)	Non-interventional, observational study	1,088 (6–12 years)	Acute bronchitis	All 5 dosage forms: cough syrup (*n* = 719) or cough drops (*n* = 64), cough liquid (*n* = 196), cough effervescent tablets (*n* = 38), cough lozenges (*n* = 49)—containing the ivy dry leaf extract EA 575 for 7 days	Doctor- and parent/patient-assessed efficacy and tolerability	There was a reported significant improvement in all findings and symptoms over the course of the 7-day therapy. In addition, very good tolerability and high compliance were demonstrated. All 5 EA 575	III
Schmidt et al. 2012 (69)	Two independent observational studies	257 (1 month to 13 years)	Acute bronchitis and cough	Ivy leaf extract [drug:extract ratio 2.2–2.9:1, the extraction solvent was a mixture of 98 parts of ethanol 50% (v/v) and two parts of propylene glycol] syrup or drops (1 × 2.5 ml syrup or 3 × 5 drops 0–1 years; 3 × 2.5 ml syrup or 3 × 16 drops 1–4 years; 4 × 2.5 ml syrup or 3 × 21 drops 4–10 years; 3 × 5 ml syrup or 3 × 31 drops 10–12 years) for 10 days	Clinical effects and tolerance as well as safety using a 4-step verbal rating scale (0 = not present, 1 = mild, 2 = moderate, 3 = severe)	The major symptoms, rhinitis, cough and viscous mucus, were found to be only mildly expressed or absent in 93, 94.2 and 97.7% of cases. The global effect was rated as “good” or “very good” in 96.5% of cases. Tolerability and compliance were found “good” to “very good” in 99% (syrup) and 100% (drops) of patients on completion of the study Ivy leaf extract in the form of syrup and cough drops was confirmed as an effective and well-tolerated treatment of cough in children. No clinically relevant differences in improvement of the various symptoms were observed based on the different formulation (syrup vs. drops).	III
Cwientzek et al. 2011 (56)	Double-blind, active comparator RCT	590 (2–86 years)	Acute bronchitis	Ivy leaf extract, ivy leaves with an extraction solvent of 50% (v/v) ethanol and propylene glycol (98:2), DER of the final spissum extract:2.2–2.9:1. vs. ivy leaf extract drops (12 drops 3× day 2–4 years; 16 drops 3× day 4–10 years; 24 drops 3× day >10 years) for 7 days	Change from baseline BSS to Visit 3	BSS decreased gradually and to a similar extent from day 1 to day 7 in both treatment groups. BSS decreased by approximately 4.7–4.9 points until day 7. The BSS subscales cough, sputum, rhales/rhonchi, chest pain during coughing, and dyspnoea improved to a similar extent in both treatment groups. Overall, 2.7% of patients (per group and overall) experienced an adverse event, all of which were non-serious. Fewer patients <10 years had adverse events than would have been expected from their share of the study population (*p* = 0.015).	II
Strauss-Grabo et al. 2011 (51)	Observational study	310 (11–85 years)	Common cold and cough	Ivy leaf dry extract; 2 × 25 mg tablets twice daily for 7 days	Tolerability and safety rated by means of questionnaires	Ivy leaf dry extract tablets were reported to have good to very good tolerability by both the practitioners (98.5%) and patients (96.4%), with high acceptance and compliance (rated as “good” in 98.8% of all cases).	III
Fazio et al. 2009 (71)	Prospective uncontrolled cohort, post-marketing surveillance study	9,657 [children 0–14 years (*n* = 5,181) and adolescents/adults 15–98 years (*n* = 4,476)]	Bronchitis (acute or chronic)	*Hedera helix L.* extract syrup [700 mg ivy leaves dry extract (drug–extract ratio 5–7.5:1)/100 ml] for 7 days: 0–5 years (2.5 ml 3× day, 6–12 years (5 ml 3× day), >12 y and adults (5–7.5 ml 3× day); physicians were free to change the dosage	Perception survey questions on change of bronchitis symptoms and tolerance	After 7 days of therapy, 95% of the patients showed improvement or healing of their symptoms. The safety of the therapy was very good, with an overall incidence of adverse events of 2.1% (mainly gastrointestinal disorders with 1.5%). Dried ivy leaf extract is effective and well tolerated in patients with bronchitis.	III
Pelargonium sidoides L. root extract (EPs 7630)							
Kamin et al. 2023 (61)	Open-label, active-controlled RCT	591 (1–5 years)	Acute bronchitis	EPs 7630 syrup (2.5 ml) (*n* = 403) or equivalent solution (10 drops) (*n* = 188) for 7 days	Safety: frequency, severity, and nature of AEs; vital signs; laboratory values. Outcome measures for evaluating the health status: intensity of coughing, pulmonary rales, and dyspnea, measured by BSS-ped; further symptoms of the respiratory infection; general health status according to the IMOS; satisfaction with treatment according to the IMPSS.	According to BSS-ped, >90% of the children experienced a symptom improvement or remission. Further respiratory symptoms decreased similarly in both groups. Over 80% of the children had completely recovered or showed a major improvement at day 7. Parents were “very satisfied” or “satisfied” with the treatment in 86.1% of patients in the syrup and solution groups combined.	II
Kardos et al. 2022 (54)	Meta-analysis (of 3 double-blind, placebo-controlled RCTs in pediatric AB)^a^	For the 3 included pediatric RCTs in acute bronchitis, *N* = 719 (mean 8.7–12.9 years)	Acute bronchitis	EPs 7630 solution (1–6 years: 3 × 10 drops/day; >6–12 years: 3 × 20 drops/day; >12–18 years: 3 × 30 drops/day) or EPs 7630 tablets (3 × 20 mg/30 mg) vs. placebo for 7 consecutive days	Cough intensity	Reduction in the intensity of cough by at least 50% of baseline values at day 7 [meta-analysis rate/risk ratio (RR), EPs 7630/placebo: 1.86 [95% CI: 1.34, 2.95], and 18.0% vs. 5.5% presented with complete remission of cough [RR: 2.91; 95% CI: 1.26, 6.72].	I
Yao et al. 2022 (34)	Meta-analysis of 7 RCTs in AB (of which 3 double-blind, placebo-controlled RCTs were in children with AB)^a^	For the 3 included pediatric RCTs in acute bronchitis, *N* = 819 (mean age ranged from 8.7 ± 4.8–12.9 ± 3.7 years)	Acute bronchitis	EPs 7630 solution (1–6 years: 3 × 10 drops/day; >6–12 years: 3 × 20 drops/day; >12–18 years: 3 × 30 drops/day) vs. matched placebo or EPs 7630 tablets (3 × 20 mg/30 mg) va placebo for 7 consecutive days	Change of BSS on day 7	See outcomes for Kamin et al., 2010a, 2010b and Kamin et al., 2012 (58–60). EPs 7630 significantly decreased the BSS on day 7 compared with the control group [*I*^2^ = 83.2%; WMD = −2.11; 95% CI: −2.65, −1.58; *p* < 0.001]. Subgroup analysis was performed based on age (adults or children and adolescents). In each subgroup, results showed that the flavonoids group significantly decreased the BSS on day 7 vs. the control group [*I*^2^ = 0%, WMD = −2.66; 95% CI: −2.99, −2.33; *p* < 0.001; and *I*^2^ = 79.9%, WMD = −1.58; 95% CI: −2.2, −0.95; *p* < 0.001; respectively)	I
Seifert et al. 2019 (12)	Meta-analysis of 6, double-blind, placebo-controlled RCTs (3 double-blind, placebo-controlled trials in AB and 3 in ATP)^a^	For the 3 included pediatric RCTs in acute bronchitis, *N* = 178 (6–10 years)	ATP (*n* = 345) or acute bronchitis (*n* = 178)	EPs 7630 solution: 1–6 years: 3 × 10 drops/day; >6–12 years: 3 × 20 drops/day; >12–18 years: 3 × 30 drops/day) vs. matched placebo or EPs 7630 tablets (3 × 20 mg/30 mg) vs. placebo *n* = 258 for 6/7 days	Cumulative paracetamol administration; inability to attend school at day 6/7	See outcomes for Kamin et al. and Kamin et al. (58–60). Compared to placebo, EPs 7630 reduced the cumulative dose of paracetamol in 5 out of the 6 trials, by an average of 244 mg [Hedges’ g; −0.28; 95% CI: −0.53, −0.02; *p* < 0.03]. At treatment end, 30.2% (EPs 7630) and 74.4% (placebo) of the children were still unable to attend school [risk ratio: 0.43; 95% CI: 0.29; 0.65; *p* < 0.001). EPs 7630 reduces the severity and duration of disease-associated symptoms in children with non-streptococcal ATP and AB. Children treated with EPs 7630 required less paracetamol co-medication and were able to return to school earlier. EPs 7630 ameliorated the unpleasant effects of the febrile response, achieved symptomatic improvement and accelerated the restoration of normal functioning without potentially counterproductive antipyresis, also reducing patient risk by avoiding the potentially harmful side effects of paracetamol.	I
Anheyer et al. 2018 (31)	Systematic review and meta-analysis of 5 RCTs in pediatrics (5 studies in the meta-analysis investigated the effectiveness of Pelargonium sidoides L.; 1 trial in tonsillitis, 1 trial in ARTI plus 3 trials *in AB*)^a^	For the 3 included pediatric RCTs in acute bronchitis, *N* = 819 (1–18 years)	Acute bronchitis (3 studies)] and RTI with an existing chronic disease (1 study) [URTI: tonsillitis (1 study)]	EPs 7630 solution (1–6 years: 3 × 10 drops/day; >6–12 years: 3 × 20 drops/day; >12–18 years: 3 × 30 drops/day) vs. matched placebo or EPs 7630 tablets (3 × 20 mg/30 mg) for 7 consecutive days	Total score of BSS rated by investigator; response rates	Meta-analysis of 5 included studies revealed evidence for efficacy of EPs 7630 in treating URTI and LRTIs symptoms [RR = 2.56; 95% CI: 1.54–4.26; *P* < 0.01; heterogeneity: *I*^2^ = 38%; *χ*^2^ = 9.63; *P* = 0.14; NNT: 8; 95% CI: 5–20) compared to placebo. Meta-analysis of four studies investigated the safety/tolerability of EPS 7630. Compared to placebo, no differences between the groups with regard to reported adverse events were observed [RR = 1.06; 95% CI: 0.42, 2.66; *p* = 0.9; heterogeneity: *I*^2^ = 72%; *χ*^2^ = 10.64; *p* = 0.01]. Moderate evidence for the efficacy and safety in the treatment of respiratory tract infections in children was reported.	I
Matthys et al. 2016 (55)	Systematic review and meta-analysis of 8 double-blind, placebo-controlled RCTs (3 of which were trials in pediatric AB)^a^	For the 3 RCTs in pediatric acute bronchitis, *N* = 819 (1–18 years)	Acute bronchitis	EPs 7630 solution (1–6 years: 3 × 10 drops/day; >6–12 years: 3 × 20 drops/day; >12–18 years: 3 × 30 drops/day) vs. matched placebo or EPs 7630 tablets (3 × 20 mg/30 mg) for 7 consecutive days	Total score BSS change during study period	See outcomes for Kamin et al. and Kamin et al. (58–60). Significant differences favoring EPs 7630 were observed for complete symptom recovery at day 7. The recovery rate under EPs 7630 exceeded that in the placebo group by more than factor 4 in children and adolescents. EPs 7630 was significantly superior to placebo in all investigated age subsets for symptom recovery from coughing [point estimate and 95% CI: 1–5 y = 4.5 (1.0; 19.9) and 6–18 y = 3.8 (1.3; 11.4)] and sputum production [point estimate and 95% CI: 6–18 y = 1.6 (1.3; 1.9)] showed a shorter time until the onset of a meaningful treatment effect and was associated with a shorter disease-related period off work, school, or kindergarten [point estimate and 95% CI: 1–5 y = 4.2 (1.2; 14.4) and 6–18 y = 2.4 (1.2; 4.9)]. Children and adolescents used less paracetamol when treated with EPs 7630, with significant differences vs. placebo in the subset under 6 years of age [point estimate and 95% CI: 1–5 years −0.6 )−1.1; −0.02) and 6–18 years −0.3 (−0.6; 0.1)].	I
Timmer et al. 2013 (57)	Cochrane review and meta-analysis of 3 double-blind RCTs in AB in children^a^	For the 3 RCTs in pediatric acute bronchitis, *N* = 819 (1–18 years)	Acute bronchitis	EPs 7630 solution (*n* = 214) (1–6 years: 3 × 10 drops/day; >6–12 years: 3 × 20 drops/day; >12–18 years: 3 × 30 drops/day) vs. matched placebo (*n* = 206) or EPs 7630 tablets (*n* = 298) (3 × 20 mg/30 mg) or placebo (*n* = 101) for 7 consecutive days	Failure to recover by day seven (complete resolution of all symptoms)	See outcomes for Kamin et al. (58–60). Subgroup analyses showed some efficacy for the liquid preparation (complete resolution of all symptoms, RR 0.82 [95% CI: 0.77, 0.88]; cough RR 0.82 [95% CI: 0.76, 0.88] [Kamin et al. (60) Kamin et al. (58)], but not for the tablet preparation [complete resolution of all symptoms, RR 0.96 (95% CI: 0.89, 1.03); cough RR 0.96 (95% CI: 0.86, 1.07); sputum RR 0.87 (95% CI: 0.71, 1.06) Kamin et al. (59)]	I
Kamin et al. 2012 (58)	Double-blind, placebo-controlled RCT	220 (1–18 years)	Acute bronchitis	EPs 7630 solution *n* = 111 (1–6 years: 3 × 10 drops/day; >6–12 years: 3 × 20 drops/day; >12–18 years: 3 × 30 drops/day) vs. placebo *n* = 109 for 7 consecutive days	Decrease of BSS from baseline (day 0) to day 7	Decrease in the BSS total score was significantly higher for children in the EPs 7630 group compared to placebo (change day 0–day 7: 4.4 ± 1.6 vs. 2.9 ± 1.4 points, respectively; *p* < 0.0001.	I
Kamin et al. 2010 (59)	Double-blind, placebo-controlled, multicentre RCT	399 (6–18 years)	Acute bronchitis	EPs 7630 film-coated tablets (1–6 years *n* = 100: 3 × 10 mg/day; > 6–12 y *n* = 99: 3 × 20 mg/day; > 12–18 y *n* = 99: 3 × 30 mg/day) or placebo *n* = 101 daily	Decrease of BSS from baseline (day 0) to day 7	The decrease in the BSS total score was more pronounced in the EPs 7630 groups than in the placebo group [placebo: 3.3 ± 2.6 points, EPs 7630 (30 mg): 3.6 ± 2.4 points, EPs 7630 (60 mg): 4.4 ± 2.4 points, EPs 7630 (90 mg): 5.0 ± 1.9]. EPs 7630 60 mg and 90 mg groups showed statistical significance: *p* = 0.0004 and *p* < 0.0001, respectively).	I
Kamin et al. 2010 (60)	Double-blind, placebo-controlled, multicentre RCT	200 (1–18 years)	Acute bronchitis	EPs 7630 solution *n* = 103 (1–6 years: 3 × 10 drops/day; >6–12 years: 3 × 20 drops/day; >12–18 years: 3 × 30 drops/day) or placebo (*n* = 97) for 7 consecutive days	Decrease of BSS from baseline (day 0) to day 7	The mean BSS score improved significantly in the EPs 7630 compared with placebo group (3.4 ± 1.8 vs. 1.2 ± 1.8 points, respectively; *p* < 0.0001).	I
Matthys et al. 2007 (73)	Prospective, open-observational, multicenter observational study	2,099 (0–93 years); children *n* = 498	Acute bronchitis	EPs 7630 solution (≤6 years: 3 × 10 drops/day; >6–12 years: 3 × 20 drops/day; >12 years: 3 × 30 drops/day) for 14 consecutive days	Change of BSS from baseline to last observation	In the subgroup for children aged 3–18 years, the mean BSS decreased from 6.3 ± 2.8–0.9 ± 1.8 at the last follow-up, and from 5.2 ± 2.5–1.2 ± 2.1 in infants aged <3 years at the last follow-up. EPs 7630 was effective and well tolerated in children and infants.	III
Haidvogl and Heger 2007 (70)	Prospective, open-observational study	742 (0–12 years)	Acute bronchitis o chronic bronchitis exacerbations	EPs 7630 solution (≤2 years: 3 × 5 drops/day; 2–6 years: 3 × 10 drops/day; >6 years: 3 × 20 drops/day) for 14 days	Change in BSS score	Overall BSS score decreased from 6.0 ± 3.0 points at baseline to 2.7 ± 2.5 points after 7 days) and 1.4 (2.1 points after 14 days). Adverse events were minor and transitory. EPs 7630-solution was shown to be a safe and an effective treatment option for acute bronchitis or acute exacerbations of chronic bronchitis in children.	III
Phytomedicine combinations							
Eucalyptus oil combination:							
Paul et al. 2010 (38)	Partially double-blind, placebo-controlled RCT	138 (2‒11 years)	URTI and nocturnal cough	Ointment with a combination of camphor, menthol, and eucalyptus oil (5 ml 2–5 years and 10 ml 6–11 years) vs. petrolatum ointment and no treatment for one single night; total study duration was two consecutive nights	Parent-reported outcome surveys of symptom relief	The ointment with camphor, menthol and eucalyptus oil was superior over petrolatum rub ointment for cough severity, child and parent sleep difficulty, and combined symptom score but not for rhinorrhea. The ointment with camphor, menthol and eucalyptus oil was not better than “no Treatment” for any outcome. In the with camphor, menthol and eucalyptus oil ointment group, 46% reported at least one adverse event vs. 0% in the petrolatum ointment and “no Treatment” groups; major events in the with camphor, menthol and eucalyptus oil ointment group were burning sensation of the skin and eyes.	II
Echinacea extract combination [from an extract of purple coneflower aerial parts and root extract, propolis and vitamin C]:							
Cohen et al. 2004 (41)	Double-blind, placebo-controlled, multicentre RCT	328 (1–5 years)	Acute RTIs	A preparation containing an extract of 50 mg/ml of echinacea (upper plant parts of *E. purpurea* and roots of *E. angustifolia*), 50 mg/ml of propolis, and 10 mg/ml of vitamin C for 12 weeks vs. placebo. Dosage was 2× daily: 5.0 ml for 1–3 years and 7.5 ml for 4–5 years. If an episode of acute illness occurred during the study, the dosage was increased to 5.0 ml and 7.5 ml, respectively, 4× daily during the episode only.	Efficacy evaluation: total no. episodes, total no. of illness days, % children with ≥1 episodes, no. episodes per child, days of illness per child and duration of individual episodes.	The number of children who experienced 1 or more respiratory tract illness episodes during the 12 weeks of the study, the total number of episodes, and the mean number of episodes per child were significantly lower in the *E.purpurea* group than the placebo group (by 43%, 55%, and 50%, respectively). The total number of illness days and duration of individual episodes were also significantly lower in the echinacea extract group compared to placebo. Similar findings were noted when the children 3 y and younger and those older than 3 y were analyzed separately (data not shown).	II
2 herb preparation [fixed combination prepared from thyme herb extract and primrose root extract]:							
Ludwig et al. 2016 (64)	Prospective cohort study	16 (1–12 years)	Acute bronchitis	2-herb fixed combination of thyme herb fluid extract and primrose root fluid extract (2.5 ml 6× day 1–4 years; 7.5 ml 6×day 5–12 years) for 7–9 days	Determination of blood ethanol concentration after oral application of the study medication; efficacy was assessed as Change in BSS (secondary outcome measure). Safety and tolerability were assessed by the investigator/patients on a global scale.	All patients exhibited a blood ethanol level below the EMA threshold of 0.125%; BSS decreased from 6.6 ± 1.0–0.9 ± 1.6 points. Global efficacy was assessed as “very good” and “good” in 60% (investigator) and 80% (patients) of cases. Global tolerability was rated as “very good” and “good” in more than 90% of cases.	III
2 herb preparation [prepared from thyme herb extract and ivy leaf extract]:							
Safina 2014 (66)	Prospective, open-label, parallel-group RCT	54 (1–6 years)	ARTI and dry cough	2-herb preparation (of thyme herb liquid extract and ivy leaf liquid extract) syrup add-on therapy (*n* = 28) vs. standard therapy only (control group)	Efficacy and safety measured and reported outcomes	An observed faster improvement of objective symptoms in the herbal combination group with significant differences (*p* < 0.05) at visit 2 (3–4 days after onset of therapy) as compared to the control group, exemplified by the number of coughing fits per day 12.1 vs. 18.3 or the rate of patients with productive cough 89% vs. 46%. The effects on these parameters were reported as excellent tolerability favoring the use of the combination as add-on therapy in the pediatric population.	III
Marzian et al. 2007 (65)	Prospective, uncontrolled cohort study	1,234 (0–17 years; <2 years (*n* = 12), 2–5 years (*n* = 372), 6–11 years (*n* = 438) and 12–17 years (*n* = 412)]	Acute bronchitis and productive cough	2-herb preparation (of thyme herb liquid extract and ivy leaf liquid extract) syrup; (2‒5 years 3 × 3.2 ml; 6‒11 years 3 × 4.3 ml; 12‒17 years 3 × 5.4 ml) per day.	Change from baseline in clinical symptoms based on BSS after 4 and 10 days of treatment.	The mean BSS score decreased from 8.8 to 4.8 after 4 days of the two herb preparation treatment and 1.8 after approx. 10 days of treatment. The frequency of coughing fits decreased by 81.3% after 10 days. As assessed by the observing physicians, the tolerability of the two herb preparation was very good or good in 96.5% of patients.	III
3-herb preparation [from a mixture of ivy leaf dry extract, marshmallow root and hedge mustard seeds]:							
Khan et al. 2019 (53)	Case-control phase 2 study	60 (10–60)	Acute cough, common cold	3-herb preparation (from a mixture of Ivy leaf dry extract, marshmallow root and hedge mustard seeds) chewable tablets for 15 days (*n* = 30 test group) vs. control group (*n* = 30)	The safety and effectiveness using the Leicester cough questionnaire (LCQ) assess cough-related QOL questions (quality of life).	At 95% CI, *p*-value was *p* ≤ 0.005 for cough variables, including cough bouts, sputum viscosity, chest congestion, sore throat and shortness of breath. The LCQ cough scale score was higher in the test group as compared to control group. The test group also showed good tolerability in terms of palatability.	III
Ali et al. 2017 (62)	Single-blind, placebo-controlled RCT	220 (3–>15 years)	Common cold, acute and chronic productive and non-productive cough, [acute URTIs: fever, post-nasal drip, body ache, sore throat, wheezing]	3 herb preparation (from the dry extract of Ivy leaf, marshmallow root and hedge mustard seeds) syrup (*n* = 110) or placebo (*n* = 110)	The severity of cough and common cold symptoms evaluated using a questionnaire before and after treatment	The 3 herb preparation syrup was very effective in the treatment of cough and common cold symptoms. In the 3 herb preparation group, 49% of patients had complete improvement, 22% moderate improvement, 16% mild improvement and 13% no improvement in symptoms. In the placebo group, 13% of patients had complete improvement, 20% had moderate improvement, 28% had mild improvement, and 39% had no improvement in symptoms.	II

AB, acute bronchitis; ACC, acetylcysteine; AE, adverse event; ATP, acute tonsillopharyngitis; BSS: bronchitis-specific symptoms/score; EMA, European Medicines Agency; IMOS, Integrative Medicine Outcomes Scale; IMPSS, Integrative Medicine Patient Satisfaction Scale; LRTI, lower respiratory tract infection; NNT, numbers needed to treat; RCT, randomized controlled trial; RTI, respiratory tract infection; 5 herb combination, *Primula veris L*. (primrose) flowers *Gentiana lutea L.* (gentian) root Verbena officinalis folium (verbena) leaves *Sambucus nigra L*. (elder) flowers *Rumex acetosa L.* (common sorrel) herb; SR, systematic review; URTI, upper respiratory tract infection; VAS, visual analog scale; WMD, weighted mean difference; y, years.

^a^ Include the same 3 RCTs (Kamin et al.) (58–60).

^b^ Includes the same 3 RCTs (Kamin et al.) and 2 cohort studies (Marzian et al. and Fazio et al.) (58–60, 65, 71).

^c^ Includes one observational study (Marzian et al.) (65).

Of the included reports for AURTIs and ALRTIs, seven reports (three meta-analyses/systematic reviews (12, 31, 34), one double-blind, placebo-controlled randomized trial (41), one partly double-blind placebo-controlled trial (38), one observational study (51) and one case-controlled study (53) contained relevant data on phytopharmaceuticals for the treatment of both AURTIs and ALRTIs. Both primary research (randomized trials and other types of studies) and secondary research (systematic reviews and meta-analyses) were included to capture essential information on efficacy and safety while providing a broader context through summarised findings. Therefore, the overall number of full text reports meeting the eligibility criteria for evaluating the efficacy and tolerability of phytopharmaceuticals for the treatment of children with URTIs and/or LRTIs combined was 45.

Below is a summary of the included studies by phytopharmaceutical group and indication (AURTI or ALRTI).

### 3.2 Preparations from *Echinacea purpurea L*. (purple coneflower)

A total of three reports (a systematic review/meta-analysis in AURTIs; one double-blind placebo-controlled randomized controlled trial; and one triple parallel-group randomized trial in AURTIs) could be evaluated for evidence of efficacy and tolerability of preparations from *Echinacea purpurea L.* for the treatment of children with AURTIs. In these reports, indications reporting on the efficacy and tolerability of preparations from *Echinacea purpurea L.* in treating symptoms of AURTI included common cold symptoms, otitis media, and unspecified ARTIs in a total of 1,498 children (Table 3).

#### 3.2.1 Evidence in AURTIs

A double-blind, placebo-controlled randomized trial by Taylor et al. (36) conducted in 407 children showed no difference in AURTI symptom severity between *Echinacea purpurea L.* dried herb extract and placebo. A systematic review by Anheyer et al. identified four randomized controlled trials investigating the efficacy of various preparations from above-ground plant parts and/or roots, which, however, could not be subjected to a meta-analysis (Table 3) due to the heterogeneity of outcome measures and control conditions of these studies (31). A triple parallel-group, randomized trial in children with common cold symptoms compared an Echinacea preparation in combination with usual treatment, a herbal preparation containing *Andrographis paniculata N.* (green chiretta) in combination with usual treatment, and usual treatment alone (42). The results of the study indicated that all three groups experienced improvements in symptoms throughout the treatment period. However, children additionally receiving Echinacea also recovered faster from common cold symptoms compared to those who only received the usual treatment, although the authors did not specifically compare the effectiveness of Echinacea in these two groups (42).

#### 3.2.2 Evidence in ALRTIs

No studies were available to evaluate the evidence of efficacy and tolerability of preparations from *Echinacea purpurea L*. for the treatment of children with ALRTIs.

### 3.3 *Hedera helix L.* (ivy) leaf dry extracts

A total of eight studies could be evaluated for evidence of the efficacy and tolerability of ivy leaf dry extracts for AURTIs and ALRTIs in children (Tables 3, 4): one double-blind, active comparator trial (56); two prospective cohort studies (63, 71); one open-label, non-interventional study (72); and four observational studies (51, 67–69); of note, one of these observational studies investigated the effectiveness of ivy leaf dry extract drops or syrup, with each treatment group evaluated as an independent non-interventional observational study (69).

#### 3.3.1 Evidence in AURTIs

One observational study could be evaluated as evidence of the efficacy and tolerability of preparations from ivy leaf dry extracts for the treatment of children with AURTIs (51). An observational study published by Stauss-Grabo et al. showed positive results regarding the tolerability and safety of film-coated tablets containing ivy extract (cough tablets) for treating the common cold accompanied by coughing (51). However, results for children could not be separated from the overall group that also included adults (51).

#### 3.3.2 Evidence in ALRTIs

A double-blind, active-controlled randomized trial published by Cwientzek et al. confirmed the safety/tolerability profile of ivy leaf, showing comparable beneficial effects of two different ivy leaf dry extract preparations on acute bronchitis in children (56).

One open-label, non-interventional study by Schönknecht et al. reported improvement in cough, chest pain on coughing, wheezing and dyspnea, auscultation changes and decline or normalization of body temperature in the majority of pediatric participants receiving a specific ivy leaf dry extract preparation for seven days (72). A prospective study by Fazio et al. indicated that ivy leaf dry extract might relieve cough and expectoration in acute and chronic bronchitis in children (71). This study also reported that ivy leaf dry extract treatment in children is well tolerated and shows a favorable safety profile (71). A prospective study by Kruttschnitt et al. (63) in children with acute bronchitis and an observational study by Olszanecka-Glinianowicz et al. (68) in children with productive cough also both reported improvement of symptoms using ivy leaf dry extract syrup. A specific ivy leaf dry extract given for ten days effectively reduced major acute bronchitis and cough symptoms in two pediatric observational studies on 257 children reported by Schmidt et al. (69).

A non-interventional, observational study by Lang et al. was conducted to assess the effectiveness and tolerability of five different dosage forms containing the ivy extract EA 575® in over 1,000 school children (ages 6–12 years) with acute bronchitis (67).The children's symptoms and overall condition significantly improved over the course of a seven-day therapy, and the dosage forms were well-tolerated with high compliance (67). All five EA 575 dosage forms were found to provide an effective and safe treatment for acute bronchitis in this group of children (67).

As mentioned above, the observational study published by Stauss-Grabo et al. demonstrated positive efficacy and safety results in children and adults taking film-coated tablets containing ivy extract (cough tablets) for treating the common cold accompanied by coughing (51). However, results for children could not be separated from the overall group that also included adults (51).

### 3.4 *Pelargonium sidoides L.* (African geranium) root extract (EPs® 7630)

A total of 20 reports were included showing evidence of EPs 7630 for the treatment of AURTIs and ALRTIs in children. Notably, three of the reports are included for both AURTI and ALRTI indications (12, 31, 34).

A total of 11 reports are included as evidence of clinical efficacy and tolerability of EPs 7630 in children with AURTI [acute tonsillopharyngitis (ATP) or sore throat]: three double-blind, placebo-controlled randomized trials (35, 39, 40); one systematic review of two randomized trials in children with tonsillitis or ARTIs (31); four meta-analyses of double-blind, placebo-controlled randomized trials (12, 13, 33, 34); one prospective, open randomized pilot study (48); and two prospective, open-label, non-controlled multicenter studies (49, 50). Notably, three of the meta-analyses evaluated varying aspects of the same three double-blind, placebo-controlled randomized controlled trials, which were Timen et al., Bereznoy et al., and Bereznoy et al. (35, 39, 40) (Table 3). A fourth meta-analysis by Kamin et al. (33) evaluated two double-blind, placebo-controlled, randomized controlled trials reported by Bereznoy et al. (35, 40).

A total of 12 reports are included as evidence of clinical efficacy and tolerability of EPs 7630 in children with ALRTI (acute bronchitis): three double-blind, placebo-controlled randomized trials (58–60); one open-label, active-controlled, randomized trial (61); one Cochrane review and meta-analysis (57); five meta-analyses and systematic reviews of various aspects of the same three double-blind, placebo-controlled trials in pediatric acute brocnhitis (12, 31, 34, 54, 55); and two prospective, open, observational studies (70, 73) (Table 4).

#### 3.4.1 Evidence in AURTIs

Three double-blinded, placebo-controlled randomized trials—Bereznoy et al., Timen et al. and Bereznoy et al.—investigated the administration of EPs 7630 to children suffering from ATP (35, 39, 40). Bereznoy et al. reported that treatment with EPs 7630 reduced the severity of symptoms and shortened the duration of illness by at least two days (35). Similarly, Timen et al. and Bereznoy et al. provide evidence that EPs 7630 is superior compared to placebo for the treatment of acute tonsillopharyngitis in children and is well tolerated (39, 40).

The identified meta-analyses reported by Seifert et al. and Anheyer et al. are described in Section 3.4.3 because results were pooled for AURTIs and ALRTIs indications (12, 31). Two more meta-analyses reported by Seifert et al. and Yao et al. included the three above-mentioned double-blind, placebo-controlled trials in children with ATP (13, 34). The analysis by Seifert et al. showed that, compared with placebo, EPs 7630 reduced the cumulative paracetamol dose by an average of 449 mg [95% confidence interval (CI): 252, 646 mg; *p* < 0.001] (13). A total of 19.1% (EPs 7630) and 71.5% (placebo) of children with ATP were still unable to attend school at the end of the treatment. According to the authors, the meta-analysis results demonstrate that EPs 7630 reduces the use of antipyretic comedication and accelerated recovery in children with ATP (13). Similarly, the meta-analysis by Yao et al. showed that EPs 7630 significantly decreased the TSS on day seven compared with the control group with no heterogeneity (34). This meta-analysis pooled the above mentioned three double-blind, placebo-controlled RCTs to analyze the primary outcome measure change of tonsillitis severity score (TSS) on day seven compared to baseline in patients with acute non-streptococcal tonsillopharyngitis. In the EPs 7630 group, the TSS was significantly decreased on day seven compared with the control group (*p* < 0.001). In addition, the complete improvement rate of symptoms, including fever, headache, difficulty in swallowing, sore throat, salivation, and pharyngeal erythema was significantly increased in the EPs 7630 group vs. the control group on day four (*p* < 0.001 for all). Pooled results of these three RCTs (35, 39, 40) with 345 children also showed that the incidence of adverse events did not differ between EPs 7630 and placebo groups (34).

A meta-analysis by Kamin et al. (33), which included the two double-blind, placebo-controlled randomized trials by Bereznoy et al. (35) and Bereznoy et al. (40), reported significantly pronounced improvement of the symptom “sore throat” after four days compared to placebo treatment, and a significant advantage of EPs 7630 compared to placebo for patients with complete remission of the symptom “sore throat” by day four. Therapy response rates under EPs 7630 by day four were reported to be significantly higher than those achieved under placebo. These results show that EPs 7630 effectively reduces the severity and time to remission of the symptom “sore throat” in children with acute non-streptococcal tonsillopharyngitis.

In a prospective, open, single-center randomized pilot study by Blochin and Heger (48), the efficacy and tolerability of EPs 7630 was compared to symptomatic treatment in children with acute tonsillitis. Results showed that EPs 7630 improved symptoms more rapidly, was better tolerated by the children, and was therefore concluded by the authors to be a superior choice for treating acute tonsillitis (48).

In a prospective, open-label study reported by Schapowal and Heger (49), EPs 7630 effectively relieved or improved symptoms of acute sinusitis in adults and children. Notably, a separate effectiveness analysis in children was not conducted. However, a subgroup analysis of a self- assessed health compliant questionnaire (HCQ-5) was analyzed for 30 children; the HCQ-5 score decreased from an initial 14.2 points to 6.8 points after 28 days (49).

A second prospective open-label study reported by Bereznoy et al. in over 1,000 patients (adults and children) with acute tonsillitis or chronic recurrent tonsillitis showed remission and/or improvements in symptoms for patients treated with EPs 7630, which was also well tolerated. The overall responder rate by day seven was 88.2% (50). However, results for children could not be separated from the overall group that also included adults.

#### 3.4.2 Evidence in ALRTIs

In three double-blind, placebo-controlled randomized trials [Kamin et al. (59), Kamin et al. (60) and Kamin et al. (58)], it was demonstrated that EPs 7630 treatment for seven days decreased the bronchitis symptom score in children with acute bronchitis as compared to baseline and to a significantly greater extent compared to placebo. Results from a recent open-label, active-controlled randomized trial conducted by Kamin et al. (61) in children under six years are also in line with the efficacy and safety/tolerability results reported in the three previously mentioned double-blind, randomized trials. In addition, the open-label study reported that the majority of parents (86.1%) were “very satisfied” or “satisfied” with the EPs 7630 treatment of their child (61).

A Cochrane review and meta-analysis by Timmer et al. (57) analyzed, among others, the above three double-blind, placebo-controlled trials [i.e., Kamin et al. (58–60)] and found that EPs 7630 may effectively relieve acute bronchitis symptoms in children. The authors of the meta-analysis reported, however, that the narrow spectrum of included studies could compromise the representativeness of the evidence (57).

A meta-analysis by Yao et al. (34) pooled seven RCTs investigating the efficacy and safety/tolerability of EPs 7630, which included a subgroup analysis of children and adolescents in the already mentioned three double-blinded, placebo-controlled randomized trials in children with acute bronchitis [i.e., Kamin et al. (58–60)]. Overall, the authors concluded that EPs 7630 may effectively relieve symptoms of acute bronchitis in children (and adults). The overall quality of the evidence was considered low for primary outcomes in acute bronchitis in children and adults. Still, it is not possible to extract the data on children only.

A meta-analysis by Kardos et al. (54) pooled data from the same three double-blind, placebo-controlled randomized trials in pediatric acute bronchitis [i.e., Kamin et al. (59), Kamin et al. (60) and Kamin et al. (58)]. A decrease in cough intensity was observed by at least 50% of children with EPs 7630 vs. placebo, as well as complete remission in cough for 18.0% of children in the EPs 7630 group compared to 5.5% in the placebo group.

In a meta-analysis by Matthys et al. (55) evaluating the change of BSS total score between baseline and treatment day seven in patients with acute bronchitis, five randomized trials were pooled, including the same three double-blind, placebo-controlled randomized trials in pediatric acute bronchitis [i.e., Kamin et al. (59), Kamin et al. (60) and Kamin et al. (58)]. The authors concluded that EPs 7630 is an efficacious, safe, and well-tolerated phytomedicine in managing acute RTIs, including acute bronchitis, in children, adolescents, and adults (55). A separate analysis in children only was not conducted.

In two prospective, open-observational studies [Matthys et al. (73) and Haidvogl and Heger (70)], EPs 7630 was also shown to be a safe and effective treatment option for acute bronchitis in children and infants.

#### 3.4.3 Pooled evidence in AURTIs and ALRTIs

Results for AURTIs and ALRTIs are pooled in two meta-analyses reported by Seifert et al. (12) and Anheyer et al. (31). The meta-analysis reported by Seifert et al. evaluated average cumulative paracetamol use and the number of children unable to go to school at day six (acute tonsillopharyngitis) or day seven (acute bronchitis) after start of EPs 7630 treatment compared to placebo in 523 children aged six to ten years (12). Compared with placebo, EPs 7630 reduced the cumulative paracetamol dose significantly by an average of 244 mg (*p* < 0.03) (12). A total of 30.2% (EPs 7630 group) and 74.4% (placebo group) of children with acute tonsillopharyngitis or acute bronchitis were still unable to attend school at the end of the treatment, corresponding to a meta-analysis risk ratio of 0.43 (*p* < 0.001) favoring EPs 7630. (*p* < 0.001) (12). The systematic review and meta-analysis by Anheyer et al. (31) reported separate analyses showing evidence of EPs 7630 compared to placebo in treating ARTI symptoms for efficacy, based on five pooled placebo-controlled RCTs (35, 58–60, 74) and for tolerability/safety, based on four pooled placebo-controlled RCTs (35, 58–60). The authors reported that EPs 7630 showed moderate evidence of efficacy and safety in treating AURTIs and ALRTIs (tonsillitis, acute bronchitis, ARTIs with transient hypogammaglobulinemia) in children and concluded that EPs 7630 could thus be considered an adjunctive therapy option for RTIs in children.

### 3.5 Pineapple fruit and stem extract

#### 3.5.1 Evidence in AURTIs

One report is included as evidence of pineapple fruit and stem extract for the treatment of AURTIs, namely rhinosinusitis, in children: The meta-analysis of two double-blind, randomized trials in children, adolescents, and adults (9 years–74 years) showed small but statistically significant intergroup differences that favored pineapple fruit and stem extract vs. standard treatment (antibiotics, antihistamines or analgesics) for nasal mucosal inflammation, nasal discomfort, breathing difficulty, and overall rating, but not for nasal discharge (32). However, results for children could not be separated from the overall group that also included adults.

#### 3.5.2 Evidence in ALRTIs

No reports could be evaluated for evidence of the efficacy and tolerability of pineapple fruit and stem extract for treating children with AURTIs.

### 3.6 Various fixed combination herbal preparations—evidence in AURTIs and ALRTIs

#### 3.6.1 Combined preparations with eucalyptus oil

Two studies evaluated the effectiveness and tolerability of combination products containing eucalyptus oil for symptoms of AURTIs such as acute rhinosinusitis [one observational study (52)] or nocturnal cough [one partially double-blind placebo-controlled trial (38)].

In an observational study by Karpova et al. (52), the duration of nasal vasoconstrictor use as a symptomatic therapy was reduced in children taking a preparation containing eucalyptus oil, among other ingredients [monoterpenes (orange oil)/d-limonene (lemon oil)/1,8-cineole (eucalyptus oil)/alpha-pinene (myrtle oil)] by 2.2 days vs. 3.6 days in the children from the control group.

An ointment containing camphor, menthol, and eucalyptus oil was compared with petroleum ointment and no treatment in a double-blind, randomized trial in 138 children with nocturnal cough (38). Parents rated the camphor, menthol, and eucalyptus oil ointment (applied on one evening only) as more favorable than petroleum ointment and no treatment for symptomatic relief of their child's nocturnal cough, congestion, and sleep difficulty caused by URTIs (38). Petroleum ointment alone was marginally better for no cough frequency treatment but not better for cough severity than no treatment (38). Although irritant adverse effects were more common among participants treated with the eucalyptus oil combination, child and parent sleep was reported to be improved overall compared with petroleum and no treatment (38). Notably, the study lasted for only two consecutive days with one single application of the camphor, menthol, and eucalyptus oil ointment for symptoms of RTIs in children.

##### 3.6.1.1 Echinacea extract combination [from an extract echinacea (aerial parts of Echinacea purpurea L. and roots Echinacea angustifolia), propolis and vitamin C]

In a randomized, double-blind, placebo-controlled study, Cohen et al. evaluated a herbal preparation containing echinacea extract, propolis and vitamin C (10 mg/ml) in order to assess its effectiveness in preventing RTIs in children from 1 to 5 years (of age) (41). Children receiving the combination experienced a 55% reduction in the number of illness episodes and a 62% reduction in the number of days with fever, as well as an overall decrease in the total number of days with symptomatic illness (41).

##### 3.6.1.2 Two-herb preparation [from a mixture of Echinacea purpurea L. (purple coneflower) aerial herb parts and root extract and Salvia officinalis L. (sage) leaves]

A double-blind, double-dummy controlled trial reported by Schapowal et al. (37) found that a spray of a two herb preparation containing an aerial herb and root extract from purple coneflower and the leaves from sage herb was at least as effective as over-the-counter chlorhexidine/lidocaine spray for sore throat in children (Table 3).

##### 3.6.1.3 Two-herb preparation [from a fixed combination of Thymus vulgaris L. (thyme) herb extract and Primula veris L./Primulus elatior L. (primrose) root extract]

One observational study by Ludwig et al. (64) documented the effectiveness of the fixed combination of a thyme herb extract and primrose root extract for treating acute bronchitis in children. The study reported that blood ethanol concentrations after oral administration of the ethanol-containing preparation in children from 1 to 12 years of age remain considerably below the EMA safety threshold (64).

##### 3.6.1.4 Two-herb preparation [from a mixture of Thymus vulgaris L. (thyme) herb liquid extract and Hedera helix L. (ivy) leaf liquid extract]

In an open-label, prospective, randomized, parallel-group study, a two-herb preparation with thyme herb extract and ivy leaf extract was investigated as an add-on therapy to standard medication in children with recurrent acute viral respiratory tract infections (66). The study found that during one week of treatment, the group receiving the two-herb preparation showed a faster improvement in objective symptoms compared to the control group with standard medication alone. There were significant differences in coughing fits per day and the rate of patients with productive cough, with the two-herb preparation group showing better outcomes (66). The two-herb preparation was well-tolerated, and based on the study's findings, it suggests that this preparation could be considered an effective add-on therapy for children with recurrent respiratory tract infections accompanied by dry cough (66).

A prospective, uncontrolled cohort study by Marzian et al. evaluated a two-herb preparation of thyme herb liquid extract and ivy leaf liquid extract for treating acute bronchitis and productive cough in 1,234 children (65). The study reported decreased clinical symptoms with the two-herb preparation, including cough from baseline and good tolerability in 96.5% of pediatric subjects (65).

#### 3.6.2 Three-herb preparation [from a mixture of *althea officinalis L.* [marshmallow] root, *Sisymbrium irio L.* [hedge mustard] seeds and *Hedera helix L.* [ivy] leaf dry extract]

The safety and efficacy of a three-herb preparation (chewable tablets) were evaluated for the treatment of ARTIs, including the common cold and cough, compared to control tablets (53). After 15 days of treatment, the effectiveness of the chewable tablets was assessed using a clinical trial questionnaire and a validated quality of life questionnaire. The results showed significant improvements in cough variables such as cough bouts, sputum viscosity, chest congestion, sore throat, and shortness of breath (53). The test group also had a higher cough scale score on the LCQ and was well-tolerated in terms of palatability. No side effects were reported, and the recipients of the three-herb preparation showed good compliance with the medication compared to the control group (53).

A single-blind, placebo-controlled randomized study by Khan et al. (43) reported that cough granules containing a mixture of marshmallow root, hedge mustard seed and ivy leaf powdered extract was effective in relieving AURTI symptoms in children (i.e., sneezing, stuffy nose, sore throat/chest discomfort, fatigue/weakness, post nasal drip) (43). This study also evaluated the effectiveness of this preparation in children with ALRTI symptoms, including chronic cough (Table 3).

A single-blind, randomized trial by Ali et al. evaluated syrup containing powdered extract of ivy leaf dry extract, marshmallow root and mustard seeds, and reported the three-herb preparation to be effective compared to placebo at reducing cough and common cold symptoms in children (62).

#### 3.6.3 Five-herb extract (prepared from extracts of *Primula veris L.* [primrose] flowers/*Gentiana lutea L.* [gentian] root/*Verbena officinalis folium* [verbena] leaves/*Sambucus nigra L.* [elder] flowers/*Rumex acetosa L.* [common sorrel] herb)

For AURTIs, three open-label prospective randomized studies evaluated the efficacy of a five-herb extract preparation (extracts of *primrose* flowers, gentian root, verbena leaves, elder flowers and common sorrel herb) for treating acute rhinosinusitis symptoms in children (44, 45, 47).

The open-label, prospective study published by Sen'kevich et al. compared the five-herb extract preparation (oral drops) with topical decongestants and topical decongestants plus antibiotics in 107 children with ARTIs, including acute rhinosinusitis symptoms (44). The study found that after a 14-day treatment period with the five-herb extract preparation, there was a notable decrease in nasal congestion, a reduction in nasal discharge, and a decrease in the quantity of blocked nasal passages (44). Moreover, the five-herb extract combination eliminated mucositis, which contributed to faster restoration of the drainage and ventilation function of the auditory tube, restored mucociliary clearance, improved the condition and well-being of children, improved the effectiveness of therapy and shortened the treatment time (44). A second open-label prospective study by Popovych et al. also reported positive effects with a five-herb extract syrup preparation, including a clinically significant, adequate reduction in the severity of rhinorrhea, nasal congestion and post-nasal drip, as well as a reduction in the prescription of antibiotics in children with acute rhinosinusitis (47). A third study investigated the efficacy and tolerability of a five-herb extract preparation (extract BNO 1012 syrup) in another open-label, prospective, interventional randomized study (45). The authors concluded that extract BNO 1012, in addition to standard symptomatic treatment of acute rhinosinusitis, provides a clinically significant, adequate reduction in the severity of rhinorrhea, nasal congestion and post-nasal drip and also leads to a reduction in the need for antibiotic use in children with acute rhinosinusitis (45) (Table 3).

#### 3.6.4 Seven-herb extract (prepared from a mixture of *Equisetum arvense L.* [horsetail] herb/*Achillea millefolium L.* [yarrow] herb/*Althaea officinalis L.* [marshmallow] root/*Juglans regia L*. [walnut] leaves *Taraxacum officinale FH Wigg.* [dandelion] plant greens *Matricaria recutita L.* [chamomile]/*Quercus robur L.* and *Quercus pubescens Willd* [oak bark])

The safety and tolerability of a seven-herb preparation (extract BNO 1030 solution containing a mixture of marshmallow root, chamomile flowers, horsetail herb, walnut leaves, yarrow herb, oak bark, and dandelion herb) plus standard antipyretic symptomatic therapy was evaluated in one open-label, randomized trial vs. standard therapy in 238 children with acute non-bacterial tonsillitis (46). The authors reported that the extract preparation of the seven-herb mixture plus standard symptomatic therapy alleviated local and general symptoms starting from day two of the disease compared with standard therapy alone. Moreover, adding the seven-herb mixed extract to standard antipyretic therapy enabled a withdrawal of antipyretics from day four (46) (Table 3).

No reports could be evaluated for evidence of efficacy and tolerability of the seven-herb extract preparation for the treatment of children with ALRTIs.

## 4 Discussion

Overall, nine of the 45 reports included were double-blind, placebo-controlled trials. Of these, six RCTs investigated the treatment of ARTIs with EPs 7630 in children. EPs 7630 was the only phytopharmaceutical with sufficient evidence to make a strong recommendation for the treatment of ARTIs in children (level of evidence/grade of recommendation: IA). One double-blind, placebo-controlled trial indicated that an Echinacea extract preparation was not effective in pediatric AURTIs. However, in contrast, one double-blind, placebo-controlled randomized study suggested the possible efficacy of an Echinacea extract combination (from an extract of purple coneflower aerial parts and root extract, propolis and vitamin C) in ARTIs (level of evidence/grade of recommendation: IIC). Weak evidence from one partially double-blind, placebo-controlled trial suggested that a camphor, menthol and eucalyptus oil ointment may help relieve sleep disturbances and reduce symptoms in children with RTIs (level of evidence/grade of recommendation: IIC). In addition to the nine double-blind, placebo-controlled trials, one double-blind, active-controlled randomized study suggests possible efficacy and safety of ivy leaf dry extract in ALRTIs (level of evidence/grade of recommendation: IIC).

Evidence that other phytopharmaceuticals phytopharmaceutical combinations are beneficial in the treatment of pediatric AURTIs and ALRTIs is limited. A summary of the evidence and recommendations by phytomedicine is provided below.

### 4.1 *Echinacea herb* preparations

*Echinacea purpurea L.* is a medicinal herb that contains various constituents reported to have various pharmacological and clinical effects (75). Preparations from *Echinacea purpurea L.* have been reported to have immunomodulatory, antiviral, and anti-inflammatory effects that have been suggested to contribute to the observed clinical benefits in adults to lower the risk of recurrent respiratory infections (76). Overall, our literature search identified weak evidence from studies in children to recommend *Echinacea purpurea L.* preparations as an effective treatment for AURTI symptoms such as otitis media and common cold in children. Due to a complete lack of reports, it is also not possible to recommend *Echinacea purpurea L.* preparations for treating pediatric ALRTIs.

### 4.2 *Hedera helix L.* leaf dry extract

*Hedera helix L.* contains saponins that are considered to have mucolytic, spasmolytic, bronchodilatory, and antibacterial effects (77). Our research retrieved only one double-blind, active-controlled randomized study as evidence for the possible efficacy and safety of ivy leaf dry extract in ALRTIs (level of evidence/grade of recommendation: IIC). Insufficient evidence was found that ivy leaf dry extracts might effectively improve AURTI symptoms in children. Therefore, randomized controlled trials in children with URTIs and LRTIs treated with ivy leaf dry extracts are warranted.

### 4.3 *Pelargonium sidoides* root extract (EPs 7630)

The *Pelargonium sidoides* root extract EPs 7630 (Dr. Willmar Schwabe GmbH & Co. KG, Karlsruhe, Germany) is a proprietary extract from the roots of the native South African plant *Pelargonium sidoides L.*, drug extract ratio 1:8–10, extraction solvent: ethanol 11% (w/w), which is widely used to treat AURTI and ALRTI (78). It is approved as a medicinal product in numerous countries in Europe, Asia, Australia, and Central and South America for the treatment of children, adolescents, and adults (78). EPs 7630 has been shown to exhibit multiple pharmacological properties, including antiviral and immunomodulatory effects (79–83). Moreover, EPs 7630 and other Pelargonium sidoides extracts contain a variety of constituents from different compound classes, including amino acids, polyphenols, flavonoids, coumarins, vitamins, and minerals. EPs 7630 in various application forms is approved in Germany and other countries inside and outside Europe for treatment of, e.g., acute bronchitis, but also for other acute respiratory tract infections in children aged one year and over. A total of 20 reports from 45 (44.4%) are included as evidence for the efficacy and tolerability of EPs 7630 treatment for AURTIs and ALRTIs in children. The most extensive randomized clinical data in children in our study was retrieved for EPs 7630 in ARTIs (six double-blind, placebo-controlled, randomized trials). Overall, the clinical evidence strongly suggests that EPs 7630 solution is superior to placebo in reducing the severity and duration of disease-associated symptoms in children with acute non-streptococcal ATP (Table 3; level of evidence/grade of recommendation: IA). Similarly, the clinical evidence strongly suggests that EPs 7630 is an appropriate treatment to reduce the severity and duration of ARTI-related symptoms in children with acute bronchitis (Table 4; level of evidence/grade of recommendation: IA).

### 4.4 Pineapple fruit and stem extract preparation

Due to limited evidence, it is currently not possible to recommend pineapple fruit and stem extract preparations as an effective treatment for ARTIs in children.

### 4.5 Herbal preparations with eucalyptus oil

Eucalyptus oil has antitussive effects, among other benefits, and is primarily used to treat coughs, colds, and bronchitis and for symptomatic relief of common cold and catarrh of the upper respiratory tract (84). It is obtained by steam distillation and rectification from the fresh leaves or the fresh terminal branchlets of various species of Eucalyptus rich in 1,8-cineole (84). Due to limited evidence, it is currently not possible to recommend eucalyptus oil preparations as an effective treatment for ARTIs in children.

### 4.6 Other combinations of herb extracts/preparations

Although one randomized double-dummy trial indicated that the two-herb preparation with a mixture of purple coneflower aerial herb parts and root extract might help alleviate symptoms in children with sore, further randomized trials are required. Although other two-herb preparations such as a combination of thyme herb extract and ivy leaf dry extract showed promise in prospective studies, robust clinical data is lacking and randomized controlled studies in pediatric RTI populations are warranted. Similarly, evidence of the effectiveness and safety of a three herb preparation from a mixture of ivy leaf dry extract, marshmallow root and hedge mustard seeds are lacking. Therefore, there is insufficient evidence to recommend any of the cited two- or three-herb preparations as effective treatments for symptoms of AURTIs or ALRTIs in children.

A five-herb extract (prepared from extracts of primrose flowers, *e*lder flowers, common sorrel herb, gentian root and lemon verbena leaves) is reported to have various pharmacological activities, such as secretolytic, expectorant, anti-inflammatory, diuretic, antimicrobial, antifungal, and sedative [7–10]. Overall, the clinical evidence suggests that the five-herb extract may be an effective adjunctive treatment for AURTI symptoms such as acute rhinosinusitis in children (level of evidence/grade of recommendation: IIC). Due to the paucity of evidence, however, it is currently not possible to recommend this five-herb extract as an effective treatment for ALRTIs in children.

Due to the limited clinical evidence, it is also not currently possible to recommend a seven-herb extract (prepared from a mixture of horsetail herb, yarrow herb, marshmallow root, walnut leaves, dandelion herb, chamomile flowers and oak bark) as an effective treatment for symptoms of AURTIs or ALRTIs in children.

We acknowledge that our findings may be subject to the general limitations of systematic reviews pertaining to potential incompleteness of the reviewed evidence and searched databases. Publication bias and location bias cannot be completely ruled out, including the tendency for negative trials to remain unpublished. Moreover, no formal assessment of bias or meta-analysis could be realized due to the heterogeneity of included study types and their variable quality. Although we performed a broad search for all RTIs in children, only a limited number of conditions were covered in the included clinical trials.

## 5 Conclusion

This systematically conducted, comprehensive literature review presents a comprehensive summary of the available evidence regarding the effectiveness and safety of phytopharmaceuticals for treating acute respiratory tract infections in children. The review found a lack of high-quality randomized controlled trials assessing the efficacy and tolerability of many phytopharmaceuticals in children with ARTI. Evidence for the safety and efficacy of several phytopharmaceutical preparations for the treatment of ARTIs in children are available: echinacea extract combination (from an extract of purple coneflower aerial parts and root extract, propolis and vitamin C), camphor, menthol and eucalyptus oil ointment, ivy leaf dry extract and EPs 7630. However, *Pelargonium sidoides L.* root extract (EPs 7630) had the strongest evidence in the pediatric population. Robust data from six double-blind, randomized, placebo-controlled clinical trials demonstrates that EPs 7630 administration in children might alleviate ARTI symptoms, reduce their severity, expedite functional recovery and decrease the need for paracetamol use. There remains a need for well-designed, randomized, placebo-controlled clinical trials to investigate the efficacy and safety of other phytopharmaceuticals for pediatric ARTI treatment.

## Data Availability

The original contributions presented in the study are included in the article/Supplementary Material, further inquiries can be directed to the corresponding author.

## References

[1] O'Brien K Bellis TW Kelson M Hood K Butler CC Edwards A. Clinical predictors of antibiotic prescribing for acutely ill children in primary care: an observational study. Br J Gen Pract. (2015) 65(638):e585–92. 10.3399/bjgp15x686497 26324495 PMC4540398

[2] Mbakwa CA Scheres L Penders J Mommers M Thijs C Arts ICW. Early life antibiotic exposure and weight development in children. J Pediatr. (2016) 176:105–13.e2. 10.1016/j.jpeds.2016.06.015 27402330

[3] Kourlaba G Kourkouni E Spyridis N Gerber JS Kopsidas J Mougkou K Antibiotic prescribing and expenditures in outpatient paediatrics in Greece, 2010–13. J Antimicrob Chemother. (2015) 70(8):2405–8. 10.1093/jac/dkv091 25881618

[4] Schrier L Hadjipanayis A del Torso S Stiris T Emonts M Dornbusch HJ. European Antibiotic awareness day 2017: training the next generation of health care professionals in antibiotic stewardship. Eur J Pediatr. (2018) 177(2):279–83. 10.1007/s00431-017-3055-0 29204852 PMC5758684

[5] Albayrak A Karakaş NM Karahalil B. Evaluation of parental knowledge, attitudes and practices regarding antibiotic use in acute upper respiratory tract infections in children under 18 years of age: a cross-sectional study in Turkey. BMC Pediatr. (2021) 21(1):554. 10.1186/s12887-021-03020-4 34872522 PMC8647354

[6] Mangione-Smith R Zhou C Robinson JD Taylor JA Elliott MN Heritage J. Communication practices and antibiotic use for acute respiratory tract infections in children. Ann Fam Med. (2015) 13(3):221–7. 10.1370/afm.1785 25964399 PMC4427416

[7] Stivers T. Managing patient pressure to prescribe antibiotics in the clinic. Paediatr Drugs. (2021) 23(5):437–43. 10.1007/s40272-021-00466-y 34410633 PMC8375467

[8] Cabral C Ingram J Lucas PJ Redmond NM Kai J Hay AD Influence of clinical communication on Parents’ antibiotic expectations for children with respiratory tract infections. Ann Fam Med. (2016) 14(2):141–7. 10.1370/afm.1892 26951589 PMC4781517

[9] Mammari N Albert Q Devocelle M Kenda M Kočevar Glavač N Sollner Dolenc M Natural products for the prevention and treatment of common cold and viral respiratory infections. Pharmaceuticals. (2023) 16(5):662. 10.3390/ph16050662 37242445 PMC10220542

[10] Ciprandi G Tosca MA. Non-pharmacological remedies for post-viral acute cough. Monaldi Arch Chest Dis. (2021) 92(1). 10.4081/monaldi.2021.1821 34461702

[11] Adeleye OA Bamiro OA Bakre LG Odeleye FO Adebowale MN Okunye OL Medicinal plants with potential inhibitory bioactive compounds against coronaviruses. Adv Pharm Bull. (2022) 12(1):7–16. 10.34172/apb.2022.003 35517886 PMC9012928

[12] Seifert G Brandes-Schramm J Zimmermann A Lehmacher W Kamin W. Faster recovery and reduced paracetamol use—a meta-analysis of eps 7630 in children with acute respiratory tract infections. BMC Pediatr. (2019) 19(1):119. 10.1186/s12887-019-1473-z 31014293 PMC6477747

[13] Seifert G Funk P Reineke T Lehmacher W. Influence of eps 7630 on antipyretic comedication and recovery from acute tonsillopharyngitis in children: a meta-analysis of randomized, placebo-controlled, clinical trials. J Pediatr Infect Dis. (2021) 16:122–8. 10.1055/s-0040-1722205

[14] Matthys H Heger M. Treatment of acute bronchitis with a liquid herbal drug preparation from Pelargonium Sidoides (eps 7630): a randomised, double-blind, placebo-controlled, multicentre study. Curr Med Res Opin. (2007) 23(2):323–31. 10.1185/030079906×167318 17288687 10.1185/030079906X167318

[15] Kardos P Bittner CB Seibel J Abramov-Sommariva D Birring SS. Effectiveness and tolerability of the thyme/ivy herbal fluid extract bno 1200 for the treatment of acute cough: an observational pharmacy-based study. Curr Med Res Opin. (2021) 37(10):1837–44. 10.1080/03007995.2021.1960493 34340607

[16] Mousa HA-L. Prevention and treatment of influenza, influenza-like illness, and common cold by herbal, complementary, and natural therapies. J Evid Based Complementary Altern Med. (2017) 22(1):166–74. 10.1177/2156587216641831 27055821 PMC5871211

[17] Firenzuoli F Gori L Neri D. Clinical phytotherapy: opportunities and problematics. Ann Ist Super Sanita. (2005) 41(1):27–33. 16037646

[18] Choi SH. Who traditional medicine strategy and activities. “standardization with evidence-based approaches”. J Acupunct Meridian Stud. (2008) 1(2):153–4. 10.1016/s2005-2901(09)60037-6 20633469

[19] World Health Organization (WHO). Who Global Report on Traditional and Complimentary Medicine. Geneva: World Health Organization (WHO) (2019). Available at: https://iris.who.int/bitstream/handle/10665/312342/9789241515436-eng.pdf?sequence=1 (Accessed February 2024)

[20] World Health Organization (WHO). Annex 5—Cumulative Index of Major Chemical Constituents. Monographs on Selected Medicinal Plants. Geneva: World Health Organization (WHO) (2009) 4. p. 406.

[21] Dias DA Urban S Roessner U. A historical overview of natural products in drug discovery. Metabolites. (2012) 2(2):303–36. 10.3390/metabo2020303 24957513 PMC3901206

[22] Jütte R Heinrich M Helmstädter A Langhorst J Meng G Niebling W Herbal medicinal products—evidence and tradition from a historical perspective. J Ethnopharmacol. (2017) 207:220–5. 10.1016/j.jep.2017.06.047 28668645

[23] Graham-Brown RAC Healsmith MF. From folklore to pharmacy: putting plants into practice. Clin Dermatol. (2018) 36(3):282–8. 10.1016/j.clindermatol.2018.03.002 29908569

[24] Martin D Konrad M Adarkwah CC Kostev K. Reduced antibiotic use after initial treatment of acute respiratory infections with phytopharmaceuticals- a retrospective cohort study. Postgrad Med. (2020) 132(5):412–8. 10.1080/00325481.2020.1751497 32312131

[25] Soilemezi D Leydon GM Yan R Simpson C Bell M Bostock J Herbal medicine for acute bronchitis: a qualitative interview study of Patients’ and health Professionals’ views. Complement Ther Med. (2020) 55:102613. 10.1016/j.ctim.2020.102613 33221589

[26] Meyer S Gortner L Larsen A Kutschke G Gottschling S Graber S Complementary and alternative medicine in paediatrics: a systematic overview/synthesis of cochrane collaboration reviews. Swiss Med Wkly. (2013) 143:w13794. 10.4414/smw.2013.13794 23740212

[27] Carr RR Nahata MC. Complementary and alternative medicine for upper-respiratory-tract infection in children. Am J Health Syst Pharm. (2006) 63(1):33–9. 10.2146/ajhp040613 16373463

[28] Holzinger F Chenot JF. Systematic review of clinical trials assessing the effectiveness of ivy leaf (Hedera Helix) for acute upper respiratory tract infections. Evid Based Complement Alternat Med. (2011) 2011:382789. 10.1155/2011/382789 20976077 PMC2957147

[29] Moher D Liberati A Tetzlaff J Altman DG. Preferred reporting items for systematic reviews and meta-analyses: the prisma statement. PLoS Med. (2009) 6(7):e1000097. 10.1371/journal.pmed.1000097 19621072 PMC2707599

[30] Howick J Chalmers I Glasziou P Greenhalgh T Heneghan C Liberati A Explanation of the 2011 Oxford Centre for Evidence-Based Medicine (Ocebm) Levels of Evidence (Background Document). Oxford: Centre for Evidence-Based Medicine (2011). Available at: https://www.cebm.ox.ac.uk/resources/levels-of-evidence/explanation-of-the-2011-ocebm-levels-of-evidence (Accessed August 2022)

[31] Anheyer D Cramer H Lauche R Saha FJ Dobos G. Herbal medicine in children with respiratory tract infection: systematic review and meta-analysis. Acad Pediatr. (2018) 18(1):8–19. 10.1016/j.acap.2017.06.006 28610802

[32] Guo R Canter PH Ernst E. Herbal medicines for the treatment of rhinosinusitis: a systematic review. Otolaryngol Head Neck Surg. (2006) 135(4):496–506. 10.1016/j.otohns.2006.06.1254 17011407

[33] Kamin W Lehmacher W Zimmermann A Brandes-Schramm J Funk P Seifert GJ Treatment of sore throat and hoarseness with Pelargonium Sidoides extract eps 7630: a meta analysis. Pharmadv. (2022) 4(2):88–103. 10.36118/pharmadvances.2022.33

[34] Yao J Zhang Y Wang XZ Zhao J Yang ZJ Lin YP Flavonoids for treating viral acute respiratory tract infections: a systematic review and meta-analysis of 30 randomized controlled trials. Front Public Health. (2022) 10:814669. 10.3389/fpubh.2022.814669 35252093 PMC8888526

[35] Bereznoy VV Riley DS Wassmer G Heger M. Efficacy of extract of Pelargonium Sidoides in children with acute non-group a Beta-hemolytic Streptococcus tonsillopharyngitis: a randomized, double-blind, placebo-controlled trial. Altern Ther Health Med. (2003) 9(5):68–79. 14526713

[36] Taylor JA Weber W Standish L Quinn H Goesling J McGann M Efficacy and safety of Echinacea in treating upper respiratory tract infections in children: a randomized controlled trial. JAMA. (2003) 290(21):2824–30. 10.1001/jama.290.21.2824 14657066

[37] Schapowal A Berger D Klein P Suter A. Echinacea/sage or chlorhexidine/lidocaine for treating acute sore throats: a randomized double-blind trial. Eur J Med Res. (2009) 14(9):406–12. 10.1186/2047-783x-14-9-406 19748859 PMC3351972

[38] Paul IM Beiler JS King TS Clapp ER Vallati J Berlin CM Jr. Vapor rub, petrolatum, and no treatment for children with nocturnal cough and cold symptoms. Pediatrics. (2010) 126(6):1092–9. 10.1542/peds.2010-1601 21059712 PMC3600823

[39] Timen G Zabolotnyi D Heger M Lehmacher W. Eps 7630 is effective in children with acure, non-*Β*-haemolytic streptococcal tonsillopharyngitis results of a double-blind, placebo-controlled, multicentre trial. Malay J Pediatr Child Health. (2015) 21:36–50.

[40] Bereznoy VV Heger M Lehmacher W Seifert G. Clinical efficacy and safety of liquid Pelargonium Sidoides preparation (eps 7630) in children with acute non-streptococcal tonsillopharyngitis. J Compr Pediatr. (2016) 7(4). 10.17795/compreped-42158

[41] Cohen HA Varsano I Kahan E Sarrell EM Uziel Y. Effectiveness of an herbal preparation containing Echinacea, propolis, and vitamin C in preventing respiratory tract infections in children: a randomized, double-blind, placebo-controlled, multicenter study. Arch Pediatr Adolesc Med. (2004) 158(3):217–21. 10.1001/archpedi.158.3.217 14993078

[42] Spasov AA Ostrovskij OV Chernikov MV Wikman G. Comparative controlled study of Andrographis Paniculata fixed combination, kan jang and an Echinacea preparation as adjuvant, in the treatment of uncomplicated respiratory disease in children. Phytother Res. (2004) 18(1):47–53. 10.1002/ptr.1359 14750201

[43] Khan MF Akram M Akhter N Mukhtiar M Zahid R Khan FS The evaluation of efficacy and safety of cough (ema) granules used for upper respiratory disorders. Pak J Pharm Sci. (2018) 31(6):2617–22. 30587469

[44] Sen'kevich OA Sidorenko SV Ditrikh OA. Comparative efficacy of various treatment regimens for children 2–5 years old with symptoms of acute viral rhinosinusitis. Vestn Otorinolaringol. (2021) 86(1):46–50. 10.17116/otorino20218601146 33720651

[45] Popovych VI Beketova HV Koshel IV Tsodikova OA Kriuchko TA Abaturov AE An open-label, multicentre, randomized comparative study of efficacy, safety and tolerability of the 5 plant—extract bno 1012 in the delayed antibiotic prescription method in children, aged 6 to 11 years with acute viral and post-viral rhinosinusitis. Am J Otolaryngol. (2020) 41(5):102564. 10.1016/j.amjoto.2020.102564 32593046

[46] Popovych V Koshel I Malofiichuk A Pyletska L Semeniuk A Filippova O A randomized, open-label, multicenter, comparative study of therapeutic efficacy, safety and tolerability of bno 1030 extract, containing marshmallow root, chamomile flowers, horsetail herb, walnut leaves, yarrow herb, oak bark, dandelion herb in the treatment of acute non-bacterial tonsillitis in children aged 6 to 18years. Am J Otolaryngol. (2019) 40(2):265–73. 10.1016/j.amjoto.2018.10.012 30554882

[47] Popovych VI Beketova HV. Results of a randomised controlled study on the efficacy of a combination of saline irrigation and sinupret syrup phytopreparation in the treatment of acute viral rhinosinusitis in children aged 6 to 11 years. Clinic Phytosci. (2018) 4(1):21. 10.1186/s40816-018-0082-y

[48] Blochin B Heger M. Umckaloabo Versus Symptomatische Therapie in Der Behandlung Der Akuen Angina Catarrhalis. Germany: OmniMed-Verlag-Ges (2000).

[49] Schapowal A Heger M. Eps® 7630 solution (Umckaloabo®) in the treatment of sinusitis. Zeitschrift für Phytotherapie. (2007) 28(2):58–65. 10.1055/s-2007-981621

[50] Bereznoy V Heger M Iljenko L Tischko F. Eps® 7630 bei erwachsenen und kindern mit angina tonsillaris. Zeitschrift für Phytotherapie. (2009) 30(1):6–13. 10.1055/s-0029-1213421

[51] Stauss-Grabo M Atiye S Warnke A Wedemeyer RS Donath F Blume HH. Observational study on the tolerability and safety of film-coated tablets containing ivy extract (prospan® cough tablets) in the treatment of colds accompanied by coughing. Phytomedicine. (2011) 18(6):433–6. 10.1016/j.phymed.2010.11.009 21211950

[52] Karpova EP Tulupov DA Emel'yanova MP. Use of myrtol standardized in the treatment of children with acute rhinosinusitis. Vestn Otorinolaringol. (2016) 81(1):47–50. 10.17116/otorino201681147-50 26977569

[53] Khan M Rehman H Naveed S Zaidi SF Ayaz S Owais A Chewable cough tablets with improved palatability: a comparative phase ii clinical trial. Pak J Pharm Sci. (2019) 32(1(Supplementary)):339–43. 30829213

[54] Kardos P Lehmacher W Zimmermann A Brandes-Schramm J Funk P Matthys H Effects of Pelargonium Sidoides extract eps 7630 on acute cough and quality of life—a meta-analysis of randomized, placebo-controlled trials. Multidiscip Respir Med. (2022) 17:868. 10.4081/mrm.2022.868 36051888 PMC9425964

[55] Matthys M Lehmacher W Zimmermann A Brandes J Kamin W. EPs 7630 in acute respiratory tract infections—a systematic review and meta-analysis of randomized clinical trials. J Lung Pulmonary Respir Res. (2016) 3(1):4–15. 10.15406/jlprr.2016.03.00068

[56] Cwientzek U Ottillinger B Arenberger P. Acute bronchitis therapy with ivy leaves extracts in a two-arm study. A double-blind, randomised study vs. an other ivy leaves extract. Phytomed. (2011) 18(13):1105–9. 10.1016/j.phymed.2011.06.014 21802921

[57] Timmer A Gunther J Motschall E Rucker G Antes G Kern WV. Pelargonium sidoides extract for treating acute respiratory tract infections. Cochrane Database Syst Rev. (2013) 10:Cd006323. 10.1002/14651858.CD006323.pub3 PMC11835051 24146345

[58] Kamin W Ilyenko LI Malek FA Kieser M. Treatment of acute bronchitis with eps 7630: randomized, controlled trial in children and adolescents. Pediatr Int. (2012) 54(2):219–26. 10.1111/j.1442-200X.2012.03598.x 22360575

[59] Kamin W Maydannik V Malek F Kieser M. Efficacy and tolerability of eps 7630 in patients (aged 6–18 years old) with acute bronchitis. Acta Paediatr. (2010a) 99(4):537–43. 10.1111/j.1651-2227.2009.01656.x 20070280 PMC2855831

[60] Kamin W Maydannik V Malek FA Kieser M. Efficacy and tolerability of eps 7630 in children and adolescents with acute bronchitis—a randomized, double-blind, placebo-controlled multicenter trial with a herbal drug preparation from Pelargonium Sidoides roots. Int J Clin Pharmacol Ther. (2010b) 48(3):184–91. 10.5414/cpp48184 20197012

[61] Kamin W Behre U Helm K Reling B Funk P Malek FA. Safety of Pelargonium extract eps 7630 in young children with acute bronchitis. Front Pediatr. (2023) 11. 10.3389/fped.2023.1107984 36865690 PMC9971625

[62] Ali Z Daniyal M Adhia MK Alam A Sarfaraz B Sattar A To evaluate the efficacy and safety of cofnovex plus (ema) syrup. Pak J Pharm Sci. (2017) 30(2(Suppl.)):591–6. 28650326

[63] Kruttschnitt E Wegener T Zahner C Henzen-Bücking S. Assessment of the efficacy and safety of ivy leaf (Hedera Helix) cough syrup compared with acetylcysteine in adults and children with acute bronchitis. Evid Based Complement Alternat Med. (2020) 2020:1910656. 10.1155/2020/1910656 32454850 PMC7222538

[64] Ludwig S Stier H Weykam S. Evaluation of blood alcohol concentrations after oral administration of a fixed combination of thyme herb and primrose root fluid extract to children with acute bronchitis. Drug Res (Stuttg). (2016) 66(2):69–73. 10.1055/s-0034-1398543 25823507

[65] Marzian O. Treatment of acute bronchitis in children and adolescents. Non-interventional postmarketing surveillance study confirms the benefit and safety of a syrup made of extracts from thyme and ivy leaves. MMW Fortschr Med. (2007) 149(27–28 Suppl):69–74. 17619603

[66] Safina AI. Treatment of young children with recurrent acute ­respiratory tract infections with a herbal combination of thyme herb and ivy leaf. Zeitschrift für Phytotherapie. (2015) 35:262–7. 10.1055/s-0034-1395797

[67] Lang C Staiger C Wegener T. Ivy in everyday paediatric use: administration of ea 575® to schoolchildren for the treatment of acute bronchitis. Zeitschrift für Phytotherapie. (2015) 36(5):192–96. 10.1055/s-0041-105237

[68] Olszanecka-Glinianowicz M Doniec Z Schönknecht K Almgren-Rachtan A. The herbal medicine containing of ivy leaf dry extract in the treatment of productive cough in children. Wiad Lek. (2020) 73(4):668–73. 32731694

[69] Schmidt M Thomsen M Schmidt U. Suitability of ivy extract for the treatment of paediatric cough. Phytother Res. (2012) 26(12):1942–7. 10.1002/ptr.4671 22532491

[70] Haidvogl M Heger M. Treatment effect and safety of eps 7630-solution in acute bronchitis in childhood: report of a multicentre observational study. Phytomedicine. (2007) 14(Suppl 6):60–4. 10.1016/j.phymed.2006.11.014 17184982

[71] Fazio S Pouso J Dolinsky D Fernandez A Hernandez M Clavier G Tolerance, safety and efficacy of Hedera helix extract in inflammatory bronchial diseases under clinical practice conditions: a prospective, open, multicentre postmarketing study in 9657 patients. Phytomedicine. (2009) 16(1):17–24. 10.1016/j.phymed.2006.05.003 16860549

[72] Schönknecht K Fal A Mastalerz-Migas A Joachimiak M Doniec Z. Efficacy of dry extract of ivy leaves in the treatment of productive cough. Wiad Lek. (2017) 70:1026–33. 29478973

[73] Matthys H Kamin W Funk P Heger M. Pelargonium Sidoides preparation (eps 7630) in the treatment of acute bronchitis in adults and children. Phytomedicine. (2007) 14(Suppl 6):69–73. 10.1016/j.phymed.2006.11.015 17184981

[74] Patiroglu T Tunc A Eke Gungor H Unal E. The efficacy of Pelargonium Sidoides in the treatment of upper respiratory tract infections in children with transient hypogammaglobulinemia of infancy. Phytomedicine. (2012) 19(11):958–61. 10.1016/j.phymed.2012.06.004 22809962

[75] Braun L Cohen M. Herbs and Natural Supplements: An Evidence-Based Guide Herbs and Natural Supplements: An Evidence-Based Guide. 4th ed Chatswood, NSW: Elsevier (2015).

[76] Schapowal A Klein P Johnston SL. Echinacea reduces the risk of recurrent respiratory tract infections and complications: a meta-analysis of randomized controlled trials. Adv Ther. (2015) 32(3):187–200. 10.1007/s12325-015-0194-4 25784510

[77] Lutsenko Y Bylka W Matławska I Darmohray R. Hedera helix as a medicinal plant. Herba Pol. (2010) 56(1):83–96.

[78] Matthys H Malek FA Kamin W. Eps® 7630 in acute respiratory tract infections—a systematic review and meta-analysis of randomised clinical research. Forsch Komplentaermed. (2014) 21:57–8. 10.15406/jlprr.2015.03.00068

[79] Emanuel J Papies J Galander C Adler JM Heinemann N Eschke K *In vitro* and *in vivo* effects of Pelargonium Sidoides dc. Root extract eps(®) 7630 and selected constituents against sars-cov-2 B.1, Delta ay.4/ay.117 and omicron ba.2. Front Pharmacol. (2023) 14:1214351. 10.3389/fphar.2023.1214351 37564181 PMC10410074

[80] Papies J Emanuel J Heinemann N Kulić Ž Schroeder S Tenner B Antiviral and immunomodulatory effects of Pelargonium Sidoides dc. Root extract eps® 7630 in sars-cov-2-infected human lung cells. Front Pharmacol. (2021) 12:757666. 10.3389/fphar.2021.757666 34759825 PMC8573200

[81] Roth M Fang L Stolz D Tamm M. Pelargonium Sidoides radix extract eps 7630 reduces rhinovirus infection through modulation of viral binding proteins on human bronchial epithelial cells. PLoS One. (2019) 14(2):e0210702. 10.1371/journal.pone.0210702 30707726 PMC6358071

[82] Theisen LL Muller CP. Eps® 7630 (umckaloabo®), an extract from Pelargonium Sidoides roots, exerts anti-influenza virus activity *in vitro* and *in vivo*. Antiviral Res. (2012) 94(2):147–56. 10.1016/j.antiviral.2012.03.006 22475498

[83] Michaelis M Doerr HW Cinatl J Jr. Investigation of the influence of eps® 7630, a herbal drug preparation from Pelargonium Sidoides, on replication of a broad panel of respiratory viruses. Phytomedicine. (2011) 18(5):384–6. 10.1016/j.phymed.2010.09.008 21036571 PMC7127141

[84] Horváth G Ács K. Essential oils in the treatment of respiratory tract diseases highlighting their role in bacterial infections and their anti-inflammatory action: a review. Flavour Fragr J. (2015) 30(5):331–41. 10.1002/ffj.3252 32313366 PMC7163989

[85] Guyatt GH. Grade: an emerging consensus on rating quality of evidence and strength of recommendations. Br Med J. (2008) 336(7650):924–6. 10.1136/bmj.39489.470347.AD 18436948 PMC2335261

